# Cascade enzymes within self-assembled hybrid nanogel mimicked neutrophil lysosomes for singlet oxygen elevated cancer therapy

**DOI:** 10.1038/s41467-018-08234-2

**Published:** 2019-01-16

**Authors:** Qing Wu, Zhigang He, Xia Wang, Qi Zhang, Qingcong Wei, Sunqiang Ma, Cheng Ma, Jiyu Li, Qigang Wang

**Affiliations:** 1 School of Chemical Science and Engineering, Shanghai Tenth People’s Hospital & Putuo District People’s Hospital, Tongji University, Siping Road 1239, Shanghai, 200092 China; 2 State Key Laboratory of Transducer Technology, Shanghai Institute of Microsystem and Information Technology, Chinese Academy of Science, 865 Changning Road, Shanghai, 200050 China

## Abstract

As the first line of innate immune cells to migrate towards tumour tissue, neutrophils, can immediately kill abnormal cells and activate long-term specific adaptive immune responses. Therefore, the enzymes mediated elevation of reactive oxygen species (ROS) bioinspired by neutrophils can be a promising strategy in cancer immunotherapy. Here, we design a core-shell supramolecular hybrid nanogel via the surface phosphatase triggered self-assembly of oligopeptides around iron oxide nanoparticles to simulate productive neutrophil lysosomes. The cascade reaction of superoxide dismutase (SOD) and chloroperoxidase (CPO) within the bioinspired nanogel can convert ROS in tumour tissue to hypochlorous acid (HOCl) and the subsequent singlet oxygen (^1^O_2_) species. Studies on both cells and animals demonstrate successful ^1^O_2_-mediated cell/tumour proliferation inhibition, making this enzyme therapy capable for treating tumours without external energy activation.

## Abstract (web summary)

Enzymatic reactions caused by neutrophils can cause the elevation of reactive oxygen species (ROS) in tumour tissue, Here, the authors, inspired by the neutrophils, design and test a synthetic cascade reaction which turns ROS into singlet oxygen and demonstrate the application of the designed nanoparticle

## Introduction

Cancer remains a leading cause of death, causing millions of deaths worldwide every year. Conventional clinical cancer therapies, including surgery, chemotherapy and radiotherapy, have some limitations, such as toxic side effects to normal cells, drug/radio resistance and an increased incidence of tumour re-growth^1–4^. The human innate immune system, arising from long-term evolution, has inspired a new biochemical approach for highly efficient cancer therapy that is achieved through nonspecific cytotoxic activity via molecular oxygen-dependent cell inactivation, lysozyme-associated digestion, and the release of immune cytokines^5–7^. Neutrophil-dependent cell inactivation commonly occurs via neutrophil lysosomes, which are several hundred nanometres in size, constituted with the so called azurophilic granules in conjunction with peroxidase^8–10^. The reactive oxygen species (ROS)-responsive cytotoxic activity of neutrophil lysosomes is determined by the biocatalytic system of myeloperoxidase (MPO), hydrogen peroxide (H_2_O_2_) and halide ions^11–13^. The detailed biochemical oxidizing reaction involves the initial enzymatic oxidation of halide by H_2_O_2_, such as Cl^−^, to hypochlorous acid (HOCl) and its subsequent decomposition to produce singlet oxygen (^1^O_2_) for the destruction of microorganisms^14,15^. Chloroperoxidase (CPO), a robust peroxidase from *Caldariomyces fumago* with higher resistance to the oxidative inactivation than MPO, was usually used for industrial catalysis of chloride by H_2_O_2_ to dominantly produce ^1^O_2_^16–19^. In general, the ^1^O_2_-involved biomedical therapy techniques^20–22^, the clinical photodynamic therapy (PDT) and the emerging sonodynamic therapy (SDT) are all referring to the excitation of sensitizers by light irradiation or ultrasound in presence of molecular oxygen to produce the dominant ^1^O_2_ at tumour sites for irreversible cellular damage^23–27^. Besides, researchers have also utilized inorganic nanoparticles loading drugs to stimulate the ROS and executed biofeton-like production of toxic hydroxyl radical (OH˙) for pathological-responsive chemodynamics treatment (CDT) of cancer^28,29^.

Inspired by the natural ^1^O_2_-generating strategy of neutrophil lysosomes, the cascade enzymes including the CPO and superoxide dismutase (SOD) has been firstly selected in this work. SOD is an important antioxidant enzyme widely used as the food antioxidant in industry to catalyse the conversion of ˙O_2_ˉ into H_2_O_2_, which can increase the amount of H_2_O_2_ for the further formation of ^1^O_2_ by the cascade biocatalysis of CPO. Our enzyme system provides ^1^O_2_-elevating strategy from the endogenous ROS (˙O_2_ˉ and H_2_O_2_) with the tuneable dosage, which even can cause the death of hypoxic tumour without the external energy activation.

A carrier suitable to entrap CPO and SOD should be similar to the membrane-bound lysosomes of neutrophils, which are several hundred nanometres in size. Nanogels, or hydrogel nanoparticles with particle sizes from a few tens of nanometres to several hundred nanometres, have been emerging as a versatile and viable platform for various biomedical applications, especially for biocatalytic proteins, because the gel matrix can protect enzymes from protein structural degradation and subsequent deactivation, and ensure higher loading and easy mobility of substrates to achieve an efficient catalytic effect^30–34^. In particular, hydrogels with a supramolecular structure, self-assembled from oligopeptides or small molecules by noncovalent interactions, have received significant attention as a protein-like material to mimic the extracellular matrix (ECM) in the fields of nanomedicine, catalysis, and tissue engineering^35–38^.

Finally, the multifunctional SOD/CPO-loaded nanogel system (SCNG) can thus be constructed by co-loading the cascade SOD and CPO in the supramolecular nanogel structure as a simulated neutrophil lysosome, responsively converting the relatively higher level of ROS in tumour microenvironment to ^1^O_2_ for tumour therapy. A hypothesis can thus be proposed: enzyme dynamic therapy (EDT), which takes full advantage of the enzymatic reactions in the tumour region, can controllably generate ^1^O_2_ to treat cancer. To the best of our knowledge, such a synergetic enzymatic process and treatment have not been previously reported.

## Results

### Construction and characterization of SCNGs

**Fig. 1:**
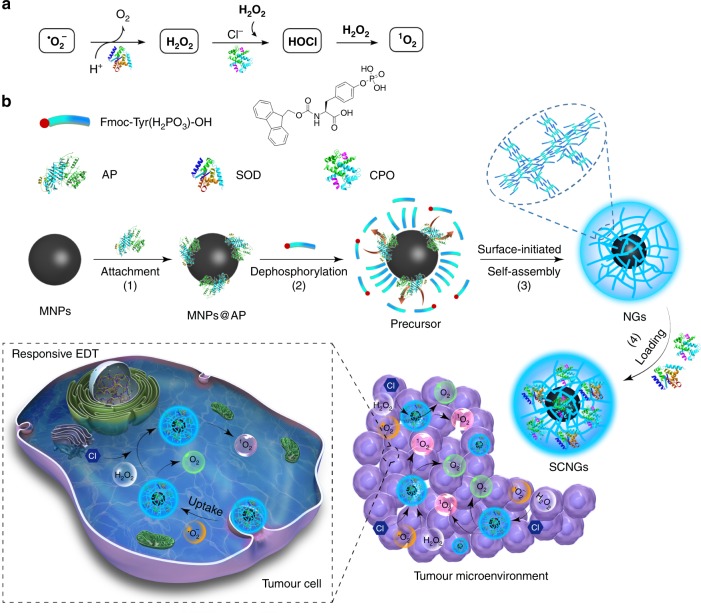
Scheme of the responsive EDT mechanism and preparation of SCNGs. **a** The cascade SOD/CPO-mediated therapy includes the catalysis of ˙O_2_ˉ into H_2_O_2_ by SOD, conversion of both as-obtained H_2_O_2_ and endogenous H_2_O_2_ species into final ^1^O_2_ species with CPO. **b** The fabrication process of SCNGs involves (1) modification of MNPs core and the AP trigger attachment, (2) dephosphorylation of hydrophilic peptide precursors to hydrophobic hydrogelators by the bounded AP, (3) AP-triggered self-assembling between the hydrophobic portions and hydrophilic part of hydrogelators through *π*–*π* stacking and electrostatic interactions around MNP, (4) immobilization of cascade SOD and CPO and further a safe and effective EDT in tumour microenvironment. The responsive SCNGs can effectively convert the endogenous ROS (˙O_2_ˉ and H_2_O_2_) into highly reactive ^1^O_2_ by the cascade reaction of SOD and CPO in tumour region, which subsequently cause cancer cells death

The proposed responsive EDT mechanism and the preparation of SCNGs are shown in Fig. 1. The EDT with cascade SOD/CPO provides a tuneable ^1^O_2_-elevating strategy by biocatalyst reaction with the endogenous ROS (˙O_2_ˉ and H_2_O_2_). It includes the catalysis of endogenous ˙O_2_ˉ into O_2_ species and H_2_O_2_ by SOD, and following conversion of both as-obtained H_2_O_2_ and endogenous H_2_O_2_ species into HClO and subsequent ^1^O_2_ species with CPO. Comparing to the commonly mentioned PDT, SDT or CDT, EDT employs the endogenous-like enzymes as the core component to cascade catalyze ROS in tumour microenvironment to drastically produce ^1^O_2_. The highly enzymatic efficiency and specificity serve this EDT the potential and unique efficacy and safety advantages. This EDT can be an efficient therapeutic tool for hypoxic tumour due to the in situ self-catalyzed O_2_ species, without any further external energy activation and with amplified ^1^O_2_ dosage due to adjustable cascade enzymes and continuous enzymatic oxidative stress (Fig. 1a). The route of SCNGs preparation process includes the synthesis of the MNP core, interface-triggered self-assembling hydrogelation and cascade enzyme encapsulations (Fig. 1b). In detail, MNP cores were synthesized and followed with carboxyl modification (MNPs@COOH) by a hydrolysis reaction with aminopropyltriethoxysilane (APTES) followed by reaction with succinic anhydride. Subsequently, the AP triggers were attached on the surface of MNPs@COOH (MNPs@AP) by amidation and further activated by 1-ethyl-3-(3-dimethylaminopropyl)carbodiimide and N-hydroxysuccinimide (EDC/NHS). Subsequently, a mild enzyme-induced assembly (EIA) technique is applied at the nanoscale surface. In particular, the bounded AP on the surface of MNPs@AP dephosphorylates the hydrophilic peptide precursors N-(fluorenyl-methoxycarbonyl) tyrosine phosphate (Fmoc-Tyr(H_2_PO_3_)-OH) to hydrophobic hydrogelators (Fmoc-Tyr-OH), which preferentially self-assemble in the aqueous solution. The inorganic nanosurface (MNPs@AP) serves as a bioactive seed layer, and the concentration of gelators is accordingly increased near the nanosurface/solution interface along with the successive enzymatic reaction. The relatively hydrophobic portions (Fmoc group) of Fmoc-Tyr-OH tend to self-assemble towards the MNP surface spontaneously to form a molecular layer, while the hydrophilic portion remains in the outside aqueous solution, and the supramolecular hydrogel can be therefore obtained by this self-assembling of Fmoc-Tyr-OH through π–π stacking and electrostatic interactions. The integrative equilibrium of enzymatic-controlled generation and deposition of gelators around the MNPs determines the thickness of supramolecular NGs.

Highly monodispersed supramolecular core-shell NGs were obtained via this AP-triggered surface-initiated self-assembling technique, and due to their magnetic MNP cores, they can be recycled by the magnetic separation method. The final simulated neutrophil lysosomes, SCNGs, were fabricated by the further immobilization of SOD and CPO onto as-obtained NGs in Tris·HCl buffer (1 M, pH 6.0) due to the hydrogen bonding between peptides and proteins. When entering a tumour microenvironment with larger oxidative stress and slightly-higher ROS levels, the SCNGs responsively catalyse the relatively high-level ROS (mainly ˙O_2_ˉ and H_2_O_2_ species) to “deadly” ^1^O_2_ species, while leaving the normal cells intact, which achieves safe and effective EDT therapy by the specifically and efficiently enzymatic generation of ROS over the threshold of the vulnerable tumour.

**Fig. 2:**
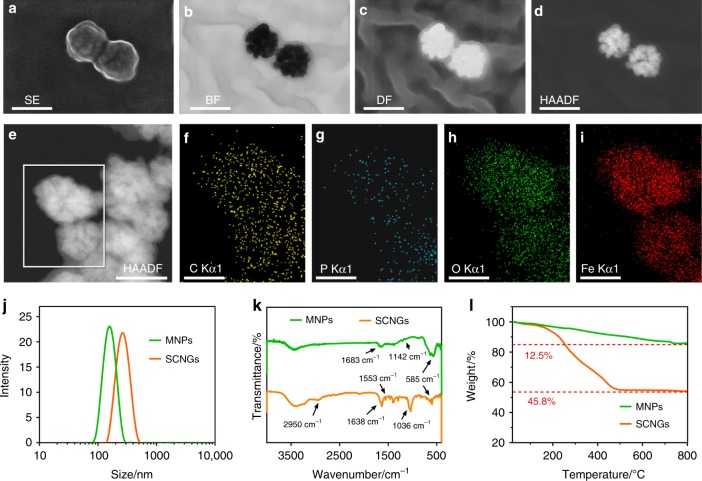
Construction and characterization of SCNGs. The STEM images of SCNGs using different modes and energy dispersive X-ray (EDX) spectroscopy mapping images of the C, P, O and Fe elements (**a** SE: second electronic, **b** BF: bright field, **c** DF: dark field, **d**, **e** HAADF: high-angle annular dark field). **j** The size distribution of MNPs (green line) and SCNGs (orange line) in buffer. **k** FT-IR spectra of the MNPs (green line) and SCNGs (orange line). **l** TG test of the MNPs (green line) and SCNGs (orange line). Scale bar in **a**–**e**, 100 nm. Scale bar in **f**–**i**, 50 nm. Source data are provided as a Source Data file

The morphology, structure and composition of the MNP core and the as-obtained SCNGs system were characterized by scanning transmission electron microscopy (STEM), energy dispersive X-ray (EDX) spectroscopy mapping, scanning electron microscopy (SEM), transmission electron microscopy (TEM) and dynamic light scattering (DLS), Fourier transform infrared spectroscopy (FT-IR) measurements and thermogravimetry (TG) analysis (Fig. 2 and Supplementary Fig. 1). Relative to the rough surface of the MNP core as the Fe_3_O_4_ cluster (Supplementary Fig. 1a, d), SCNGs exhibit more spherical structure and smooth surface with better monodispersity (Supplementary Fig. 1b, c, e, f). An obvious light-contrast organic hydrogel nanoshell can be observed around the black MNP core in STEM image under different modes, especially the BF and DF modes, as shown in Fig. 2. A further element mapping of corresponding nanogels by EDS (Fig. 2f–i), showing a spherical distribution of elemental C and P from the residual phosphate groups on the surface of MNP core, further indicate the successful coating of the supramolecular hydrogel nanoshell structure.

The average diameters of the air-dried MNP core and SCNGs observed in the STEM images are ~80 nm and 110 nm, respectively. Furthermore, the dynamic diameter distributions were further investigated by DLS measurements (Fig. 2j). The supramolecular core-shell SCNGs possess an average dynamic diameter of ~250 nm with a favourable dispensability. Usually, the hydrogel nanoparticles in the aquatic solution exhibit much higher size value in DLS test, due to a larger average hydrodynamic diameter including the immobilized water inside the hydrogel network and the hydration layer around, comparing to the dried size obtained by SEM or TEM. Comparatively, MNPs are measured to be ~150 nm, also confirming the existence of a supramolecular hydrogel structure. The component and content of organic hydrogel nanoshell were further assessed by FT-IR and TG analysis, as illustrated in Fig. 2k, l. Both the FT-IR spectra of MNPs (green line) and SCNGs (orange line) exhibit the absorption peaks of iron oxide structure at around 585 cm^−1^ (Fe–O vibrations). The characteristic bands in the region of 1683–1142 cm^−1^ of MNPs can be observed arising from the stabilized layer of polyvinyl pyrrolidone (PVP)^39^. The successful gelatin process of SCNGs based on the peptide hydrogelators can be validated by the appearance of increased intensity of -C–O–C- peak at 1036 cm^−1^ and the -CH_2_ stretching at 2950 cm^−1^, and the significantly decreased Fe–O vibrations;^40^ The increased intensity of the peaks at 1638 (ν_CO_ Amide I band) and 1553 (δ_NH_ Amide II band) in the SCNGs spectrum belong to protein amide band from SOD and CPO^41,42^. Quantitatively, a more 33.3% weight loss of lyophilized SCNGs relative to MNPs can be obtained in the TG curves. The accurate amount of peptides hydrogelator and cascade enzymes can therefore be calculated to be approximately 0.62 g per 1 g of MNPs. As a proof, the corresponding bulk hydrogel can be finally obtained by this surface modified AP-triggered interfacial hydrogelation and furtherly crosslinking nanogel to form bulk hydrogel at the higher hydrogelators (about 10 times), shown in Supplementary Fig. 2. The storage modulus (G’) in the hydrogel rheological test is higher than the loss moduli (G”) in the corresponding frequency sweep tests also indicating the successful hydrogelation process. Both the morphology and structural features as well as the composition analysis indicate that a successful supramolecular hydrogel nanoshell was constructed based on the introduced MNP core by the developed AP-triggered surface-initiated self-assembling technique.

As an enzymatic therapy mode, the stability and enzyme activity under different pH values and storage times should be evaluated. (Supplementary Figs. 3–6 and Supplementary Table 1). Firstly, the stability of SCNGs, including the release of the enzymes in PBS at 37 ℃ for 14 days, the diameter and the morphology change after being treated in cell culture media containing 10% (v/v) fetal calf serum (FCS) for 24 and 48 h have been tested, as shown in Supplementary Figs. 3–4. There is no significant release of enzymes during the 14 days, and the morphology by TEM after treatment stay intact, indicating the good stability of SCNGs without disassembly after a long time storage. While the corresponding average sizes increased from 255 nm to about 295 nm after treatment, possibly due to the absorption of serum in the culture media. Besides, the loading amount of SOD and CPO in SCNGs were detected to be 43.8 U mg^−1^ and 25.0 U mg^−1^ of NGs as illustrated in Supplementary Table 1, respectively. Their activities were evaluated based on the ability of SOD to inhibit the autoxidation of pyrogallol and CPO to catalyze the conversion of monochlorodimedon (MCD) to dichlorodimedon (DCD)^43,44^. The activities of SOD and CPO within SCNGs remained 76 and 84% of that relative to free enzymes, respectively. Furthermore, the related enzyme activities in the simulated acid intracellular environment at pH = 7.0, 6.0, 4.6 have been studied as shown in Supplementary Fig. 5. The activity of SOD in SCNGs remained ~80% at pH 4.6, while CPO exhibited 289% activity at pH 4.6 compared to the activity at pH 7.4, because of the optimum catalytic pH of CPO at about 4.5. Finally, the storage activity tests during 30 days have been also conducted (Supplementary Fig. 6). Both the activity of SOD and CPO in SCNGs did not show any significant activity loss. The supramolecular hydrogel matrix can relieve the enzyme protein degradation by the non-covalent immobilization and sacrifice protection by their protein-like structure, ensuring high structural stability and enzymatic capability for efficient catalysis^45^.

### Characterization of the ^1^O_2_ generation and efficacy

**Fig. 3:**
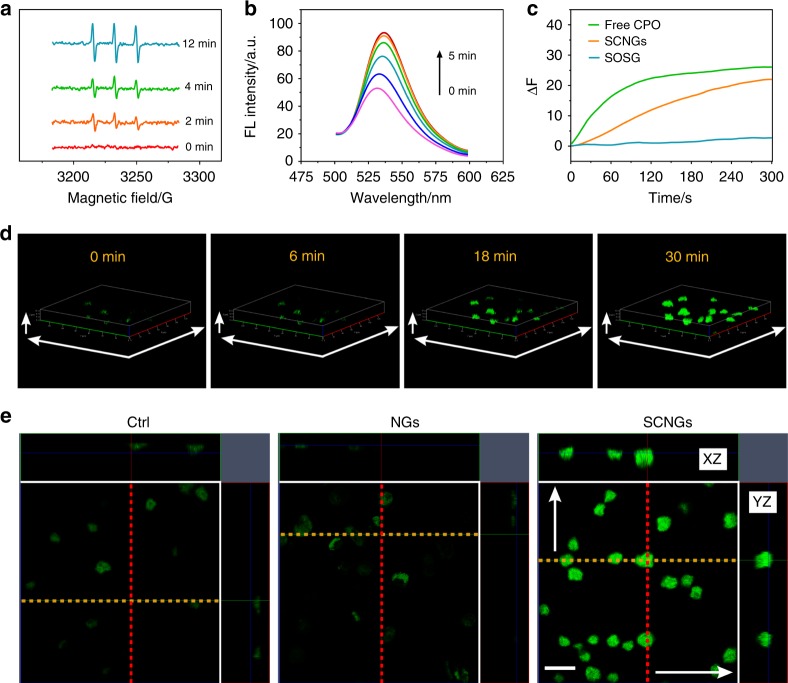
Characterization of the generation of ^1^O_2_. **a** Time-dependent electron paramagnetic resonance (EPR) signals of ^1^O_2_ from SCNGs in the presence of 2,2,6,6-tetramethylpiperidine (TEMP). TEMP was served as a ^1^O_2_ trapper. **b** Fluorescence spectra of the singlet oxygen sensor green (SOSG) with SCNGs in PBS buffer (20 mM, pH 6.8). **c** Time-dependent fluorescence spectra of SOSG with free CPO and SCNGs in PBS buffer (20 mM, pH 6.8). a.u., arbitrary units. **d** Three dimensional confocal laser scanning microscopy (3D-CLSM) images of living HepG2 cells after co-incubation with SCNGs at 300 μg mL^-1^ for 2 h and treated with SOSG probe (5 µM) from 0 min to 30 min. Scale bar, *X*-axis: 140 μm, *Y*-axis: 140 μm, *Z*-axis: 12 μm. **e** The corresponding CLSM photomicrograph with YZ (cells on the red line) and XZ (cells on the orange line) planes of living HepG2 cells and cells after co-incubation with NGs and SCNGs at 300 μg mL^-1^ for 2 h and treated with SOSG probe (5 µM) at 18 min. Scale bar, 20 μm. Source data are provided as a Source Data file

The key to effective EDT lies in the efficacy of ^1^O_2_ generation (Fig. 3). The EPR technique was employed, using TEMP as a trapping agent and H_2_O_2_ (100 µM) in NaCl solution (100 mM) as endogenous environment, to qualitatively detect and validate ^1^O_2_ signals generated by SCNGs. As shown in Fig. 3a, after mixing the SCNGs and ^1^O_2_ trapper (TEMP), a typical ^1^O_2_-induced 1:1:1 triplet signal was clearly observed in the EPR spectra (with a hyperfine splitting constant α_N_ = 17.34 G and a *g* value = 2.0056 of the photoproduct of TEMP-singlet oxygen^46^), and its intensity increased over time. A further catalysis mechanism and efficacy of cascade enzymes was demonstrated by comparing the efficacy of ^1^O_2_ generation of SOD and CPO, single SOD or CPO systems. In order to simulate the endogenous ˙O_2_ˉ and H_2_O_2_ in ROS, the catalysis of xanthine oxidase (XO, 13 U mL^−1^) and xanthine (X, 25 mM) have been introduced, which was reported to generate most of ˙O_2_ˉ species and additional small amount of H_2_O_2_^47^. As illustrated in Supplementary Fig. 7, in the SOD systems, negligible ^1^O_2_ signals generated in the spectra even in 10 min after reaction. While in the CPO catalysis system, a relatively weaker peaks of ^1^O_2_ signals were obtained by the catalysis reaction from CPO and XO/X-derived H_2_O_2_. Considerable and increased ^1^O_2_ signals in EPR spectra were observed in the SOD and CPO cascade system along with the reaction times from 0 min to 10 min, which is consistent with the ERP results of SCNGs in Fig. 3a. All the results verified that the cascade SOD and CPO in SCNGs can efficiently biocatalyze ROS in tumour tissues to the ^1^O_2_ species with tuneable dosage for anti-tumour therapy.

Furthermore, the efficacy of ^1^O_2_ generation can be evaluated by fluorescent probes, such as Singlet Oxygen Sensor Green reagent (SOSG)^48^. As illustrated in Fig. 3b, the fluorescence emission intensity of SOSG by reacting with SCNGs gradually increases as the reaction time increased from 0 min to 5 min in the H_2_O_2_ and NaCl solution, verifying the production of ^1^O_2_ by the SCNG systems with H_2_O_2_ and NaCl. The time-dependent fluorescence spectra of SOSG that reacted with SCNGs or free CPO in the presence of H_2_O_2_ and NaCl are displayed in Fig. 3c, which indicates that the ^1^O_2_ generation is increasing over time. The SCNGs here show a stable and sustaining reactive process as shown in the spectra; the activity is still considerable and favourable in the immobilized enzyme system. Relative to the free CPO, SCNGs show no significant impacts on the production of ^1^O_2_, only a slower ^1^O_2_ generation rate in the same concentration of CPO. Hence, a simulated biochemical oxidizing reaction of neutrophil lysosomes occurs during the SCNG system, in which ^1^O_2_ species are generated by the enzymatic reaction of CPO oxidation of Cl^-^ by H_2_O_2_.

In addition, the characterization of ^1^O_2_ from therapeutic agents was further evaluated on a HepG2 cell model with starvation treatment. After co-incubation with SCNGs for approximately 2 h, the fluorescent probe SOSG was added for subsequent examination by CLSM from 0 min to 30 min. As the prolonging of time (Fig. 3d), the living HepG2 cells exhibit more and more stronger green fluorescence in 3D-CLSM, indicating abundant ^1^O_2_ inside the cells originating from the enzymatic reaction with the intracellular ROS. In order to further distinguish the impacts of SCNGs and HepG2 cells themselves on the ^1^O_2_ generation, HepG2 cells were co-incubated with NGs and SCNGs at 300 μg mL^−1^ for 2 h. The CLSM photomicrograph with YZ (cells on the red line) and XZ (cells on the orange line) planes were captured after adding SOSG probe for 18 min. In addition to the control group and NGs group with negligible fluorescence signals, SCNGs group shows extensive green ^1^O_2_ signals within the whole cell from both the YZ and XZ plane imaging, as shown in Fig. 3e. All the results verify that the therapeutic agent SCNGs can significantly increase the production of ^1^O_2_ in tumour microenvironment responsively, to fulfil the potential EDT with high safety and efficacy.

### In vitro EDT

**Fig. 4:**
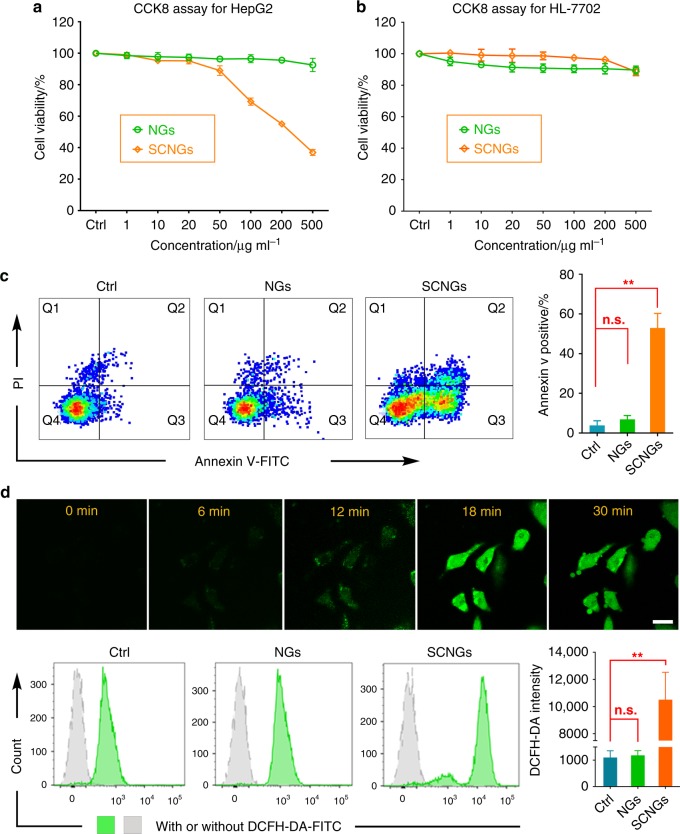
Safety, efficiency and mechanism of EDT. **a** Cytotoxicity of NGs and SCNGs by CCK8 assays after incubated with HepG2 cells for 24 h with the concentration of 1 μg mL^-1^ to 500 μg mL^-1^. NGs exhibited no significant effect on the cell survival, while SCNGs had increased cytotoxicity to HepG2 cells with the increase of concentration. Data are presented as mean ± s.d. (*n* = 3). **b** Cytotoxicity of NGs and SCNGs by CCK8 assays after incubated with HL-7702 cells for 24 h with the concentration of 1 μg mL^-1^ to 500 μg mL^−1^. Both NGs and SCNGs exhibited no significant effect on the cell survival. Data are presented as mean ± s.d. (*n* = 3). **c** The flow cytometry results of the apoptosis of HepG2 cells staining with Annexin V-FITC/PI after incubated with NGs and SCNGs at the IC_50_ value for 24 h (left), and the corresponding data analysis (right). The enhanced apoptosis promoted by SCNGs confirms the significant effect in cancer-cell killing of SCNGs. Data are presented as mean ± s.d. (*n* = 3). *p* values were analyzed by Student’s two-sided *t*-test (**P ≤ 0.01, n.s. represents no significant differences). **d** Time-dependent CLSM images (top) and corresponding flow-cytometry analysis (bottom) of HepG2 cells treated with NGs and SCNGs at the IC_50_ value for 24 h using carboxy-H_2_DCFDA as a ROS detector (left), and the corresponding data analysis (right). The experiment was conducted three times, and representative results are present. Scale bar, 20 µm. Data are presented as mean ± s.d. (*n* = 3). *p* values were analyzed by Student’s two-sided *t*-test (**P < 0.01, n.s. represents no significant differences). Source data are provided as a Source Data file

After the analysis of ^1^O_2_ species from SCNG therapeutic agents, the efficiency and safety of EDT were investigated in vitro on hepatoma carcinoma HepG2 cells and normal hepatic cells HL-7702, respectively. First, the cytotoxicity of the as-prepared NGs and SCNGs with the concentration from 1 μg mL^−1^ to 500 μg mL^−1^ was evaluated by CCK-8 assays after incubation with HepG2 cells by starvation treatment for 24 h. As shown in Fig. 4a, the NG itself shows almost no detectable cytotoxicity towards the HepG2 cells, with approximately 90% cell viability even at a concentration of 500 μg mL^−1^. By contrast, with the therapeutic ^1^O_2_ mediated by the loaded cascade SOD/CPO, SCNGs exhibits significant cell proliferation inhibition, with an IC_50_ approximately 291.24 μg mL^−1^. Besides, the safety of SCNGs was further evaluated by the cytotoxicity test with CCK8 assays after co-incubated with HL-7702 cells for 24 h with the concentration from 1 μg mL^−1^ to 500 μg mL^−1^. As shown in Fig. 4b, both NGs and SCNGs exhibited no significant effect on the HL-7702 cell survival, all the viability values are nearly at about 90% even at high concentration of 500 μg mL^−1^. In this study, SCNGs can significantly inhibit the tumour cell proliferation and conversely show negligible cytotoxicity to normal cells, indicating a high efficiency and safety of EDT in vitro.

Furthermore, the apoptosis of HepG2 cells incubated with NGs and SCNGs at the IC_50_ value for 24 h was analysed more precisely by flow cytometry after staining with annexin V-FITC/PI (fluorescein isothiocyanate/ propidium iodide). As shown in Fig. 4c, relative to the PBS group (approximately 3.9%), treatment with NGs induces no evident apoptosis (approximately 6.9%), while SCNGs significantly promote the apoptosis of the tumour cells (approximately 53%). These results indicate that SCNGs can serve as safe and efficient potential agents for tumour therapy due to their considerable biocompatibility, lack of significant toxic effects to normal cells, and (conversely) strong cytotoxicity to cancer cells with oxidative stress.

This biochemical oxidizing reaction between the SCNG system and microenvironmental ROS, which involves many oxidant products, such as ^1^O_2_ and HOCl, can further stimulate more ROS to initiate another round of enzymatic reactions by the immobilized SOD/CPO, theoretically providing high effectiveness for the tumour therapy via this developed EDT technique. Thus, as a proof-of-concept study, the intracellular ROS condition in tumour cells was investigated after 24 h of incubation with SCNGs at the IC_50_ value by using 5-chloromethyl-2′,7′-dichlorodihydrofluorescein diacetate acetyl ester (DCFH-DA) as the ROS fluorescent probe, as illustrated in Fig. 4d. An increased strong green fluorescence can be detected under CLSM from 0 min to 18 min after adding DCFH-DA probe, which indicates that the DCFH-DA probe molecules gradually enter the HepG2 cells containing elevated ROS (top of Fig. 4d). Further quantitative analysis was performed on HepG2 cells incubated with PBS, NGs or SCNGs using flow cytometry at 18 min after adding the probe (bottom of Fig. 4d). As expected, only SCNGs can effectively upregulate the level of ROS, and the NGs perform similarly to the control. For a comparative study, the endogenous ROS level of HL-7702 cells with and without SCNGs treatment have been tested (Supplementary Fig. 8). As expected, the preliminary ROS levels in HepG2 cells are slightly higher than the levels in HL-7702 cells. And the level of ROS in the HL-7702 cells treated by PBS as ctrl group and treated by SCNGs show no significant difference, that is to say the endogenous ROS in HL-7702 cells cannot trigger much enough killing ROS to induce the cell apoptosis, which can be attributed to the strong antioxidant response in normal cells comparing to the tumour cells with exhausted antioxidant response at large oxidative stress^20,49^. The intracellular ROS activity in tumour cells is greatly enhanced by the cascade reaction of SOD and CPO in SCNGs inside the cancer cells, which can serve as one of the basic principles for the highly efficient EDT.

**Fig. 5:**
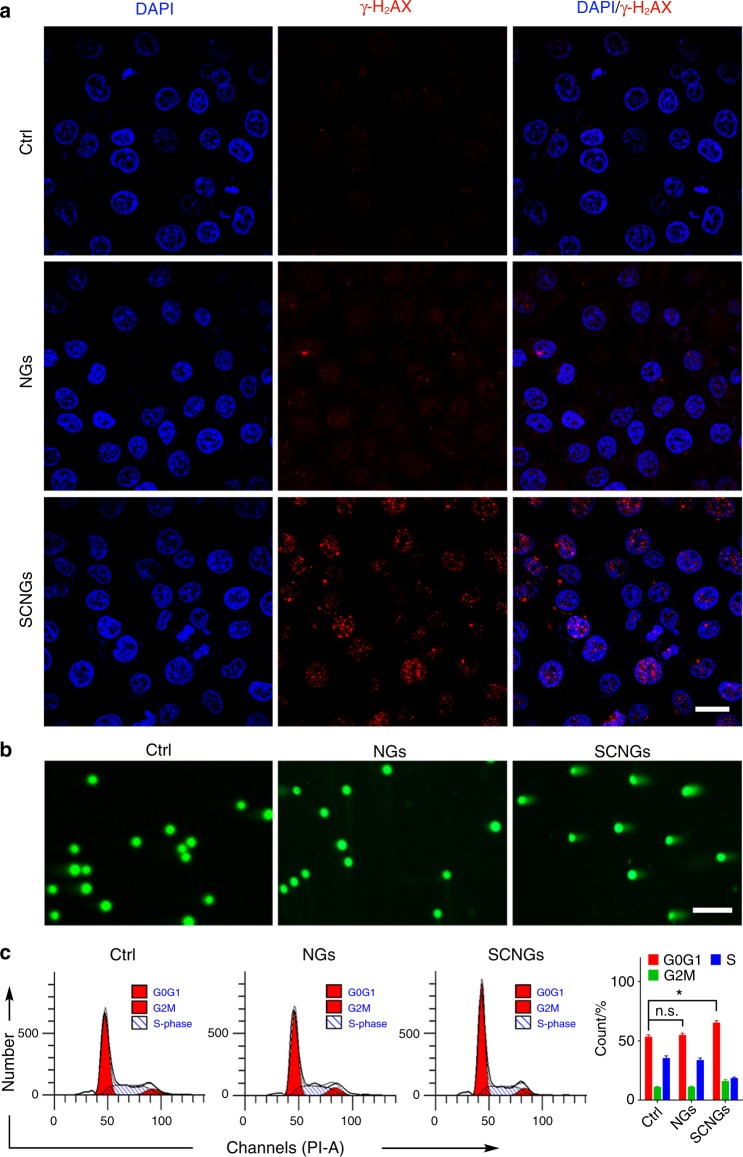
Mechanism of tumour cell apoptosis induced by EDT. **a** Representative CLSM images of different groups of HepG2 cells using γH2AX as a DNA damage biomarker. Scale bar, 20 µm. The red fluorescence indicates the DNA damage of HepG2 cells caused by ^1^O_2_ after SCMGs treatment. **b** Representative fluorescent images of DNA damages in different groups of HepG2 cells using the neutral comet assays. Scale bars, 50 μm. The appearance of longer comet tails in HepG2 cells indicates the damaged DNA doubles-strand breaks. **c** Effects of SCNGs on cell cycles by flow cytometry assays on different groups of HepG2 cells with PI staining (left), and the corresponding data analysis (right). The control, NGs and SCNGs groups are referring to the HepG2 cells after incubation with PBS, NGs and SCNGs respectively at the IC_50_ value for 24 h. The all experiments were performed three times, and the representative results are displayed. Data are presented as mean ± s.d. (*n* = 3). *p* values were analyzed by Student’s two-sided *t*-test (*P < 0.05, n.s. represents no significant differences). Source data are provided as a Source Data file

Before the study of EDT in vivo, the mechanism of tumour cell apoptosis induced by SCNGs were investigated. The γH2AX assay (phosphorylation of the core histone protein H2AX), neutral comet assays and cell cycle analysis were conducted. γH2AX is used as a marker to track the amount of DNA double-strand breaks (DSBs) to evaluate the DNA damage induced by ^1^O_2_. As shown in Fig. 5a, the red fluorescence of γH2AX remarkably increases in the SCNG group relative to the group of NGs and the control, indicating that cells exposed to SCNGs suffer DNA damage by ^1^O_2_. In order to directly visualize the DNA damages in HepG2 tumour cells, we employed the neutral comet assays, which have been reported to exclusively detect DNA doubles-strand breaks induced by various DNA-damaging agents. As shown in Fig. 5b, comet tails in HepG2 cells treated with SCNGs are significantly longer compared with the NGs and control treatments, showing much more damaged DNA doubles-strand breaks existed in SCNGs group. In addition, DNA damage usually lead to the perturbation of cell cycle progression; thus, flow cytometry assays on HepG2 cells after incubation with PBS, NGs and SCNGs respectively at the IC_50_ value for 24 h with PI staining were conducted (Fig. 5c). The SCNGs treatment results in a noticeable obstruction of the cell cycle progression and interrupts the G0/G1 checkpoint transition leading to the cell apoptosis. All these results verify that the mechanism of tumour cell death induced by EDT is the ^1^O_2_-induced DNA damage that further interferes with cell cycle progression.

### In vivo EDT

**Fig. 6:**
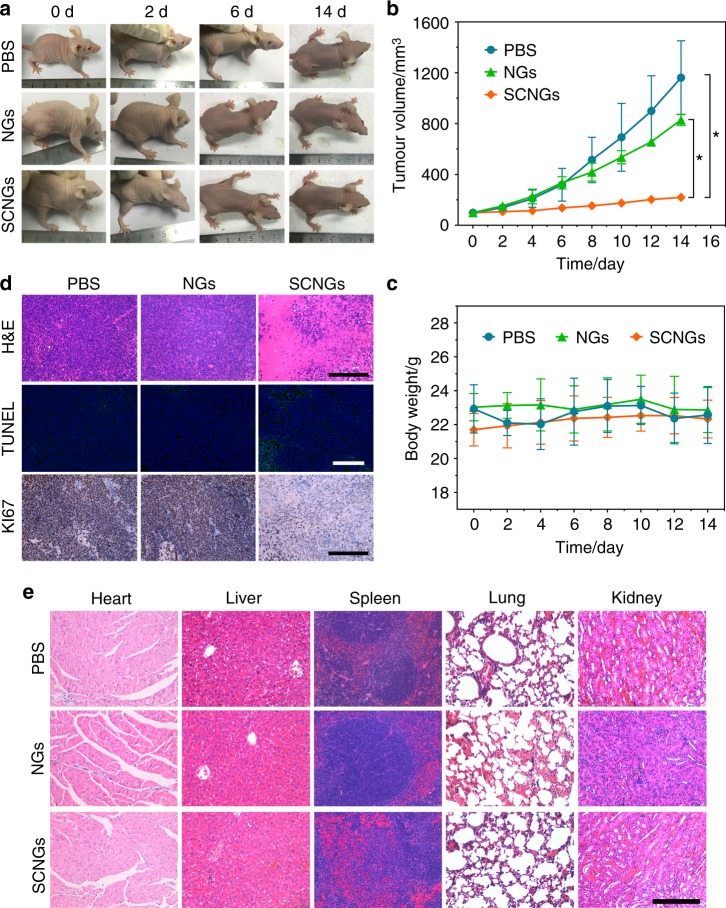
In vivo EDT on HepG2 cell derived mouse model. **a** Photographs on the 0, 2nd, 6th, 14th day of tumour-bearing mice after various treatments by intravenous injection. Dose: PBS (100 μL), NGs (100 μL, 5 mg mL^−1^), SCNGs (100 μL, 5 mg mL^−1^). The relative tumour volume (**b**) and body weight (**c**) change curves of each group of mice in 14 days after various treatments. Date are the means ± s.d. (*n* = 3). *p* values were analyzed by Student’s two-sided *t*-test (*P < 0.05). **d** Histopathology analysis (H&E staining, TUNEL and KI-67 assay) of the tumour tissue after 14 days of different treatments. Scale bar, 200 μm. **e** H&E staining of the major organs of each group of mice after 14 days of different treatments. Scale bar, 200 μm. Source data are provided as a Source Data file

At last, the nude mice bearing with HepG2 cell derived liver cancer model have been established to further evaluate the in vivo efficacy of EDT. The tumour-bearing mice with similar tumour size of 80–100 mm^3^ were divided into three groups, including PBS control group, NGs and SCNGs group, respectively. Each mouse was intravenously injected by PBS, NGs or SCNGs at 5 mg mL^−1^ of 100 μL (with the dose of 20 mg kg^−1^ mouse), followed by recording the tumour sizes using a calliper every other day to examine the EDT effects for 14 days. The tumour sizes in SCNGs group increased slightly, indicating a significant inhibition of tumour growth. By contrast, mice treated with NGs and PBS presented a fast tumour growth during the 14 days treatment for intravenous injection (Fig. 6a, b). Besides, no significant body weight changes can be also observed in all three groups for intravenous injection during the therapeutic process (Fig. 6c). Finally, the mice were sacrificed to collect the tumours and organs for histological analyses. As depicted in Fig. 6d, the pathological structure and morphology, apoptosis and proliferation of the cells in tumour sections were analysed by H&E (haematoxylin and eosin), TUNEL (Terminal deoxynucleotidyl transferase dUTP nick end labeling) and KI-67 (nuclear-associated antigen KI-67) tests. The cells in tumour sections exhibited obvious destruction with large vacuoles and irregular widening nucleus after SCNGs treatment in H&E staining, while the tumour tissue of NGs and PBS group still remained intact and undamaged. In addition, only the tumour tissues treatment with SCNGs demonstrated significant TUNEL-positive apoptotic tumour cells (green fluoresce) and decreased KI-67-positive proliferating tumour cells. All these results represent the potential high therapeutic effect of SCNGs for EDT. Moreover, to evaluate the toxicity and side effects of SCNGs in vivo, the H&E stained tissue slices of the major organs were also analysed, including heart, liver, spleen, lung and kidney, as shown in Fig. 6e. No noticeable damage or pathological change were observed in the organ slices after injection of SCNGs, which revealed that SCNGs possessed no evident side effects and can be a safe agent for further in vivo applications.

**Fig. 7:**
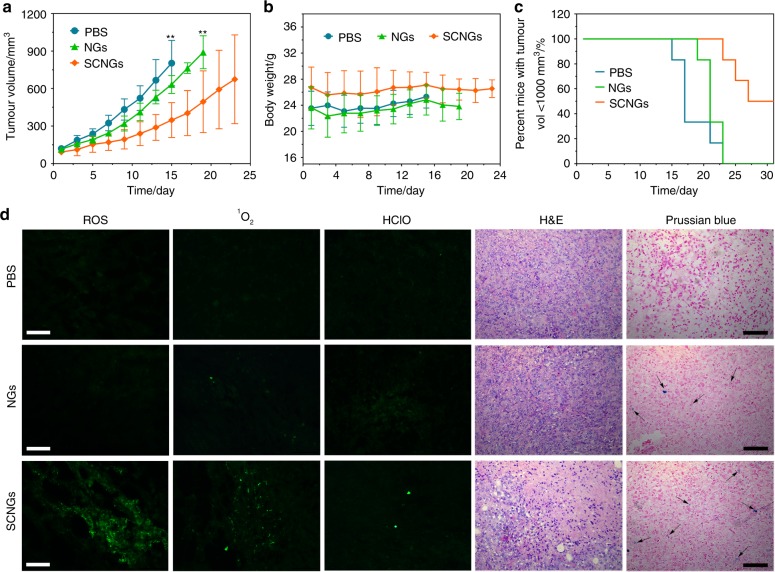
In vivo EDT on HCC PDX mouse model. The relative tumour volume (**a**), body weight (**b**) change curves and the corresponding survival percentages (**c**) of each group of mice. Dose: PBS (100 μL), NGs and SCNGs (100 μL, 5 mg mL^−1^). Date are the means ± s.d. (*n* = 6). *p* values were analyzed by Student’s two-sided *t*-test (**P < 0.01). **d** Histopathology analysis of the HCC PDX tumour tissue: ROS, ^1^O_2_, HClO, H&E and Prussian blue staining, respectively. Scale bar, 100 μm. Source data are provided as a Source Data file

To further reveal the potential therapeutic effect of SCNGs for in vivo EDT, a more relevant animal model, the Hepatocellular carcinoma (HCC) patient derived xenograft (PDX) mouse model has been employed to investigate the efficacy of anti-tumour and the corresponding survival percentages. Considering the significant differences of tumour growth due to the different sources of patient tumor tissues and genomic diversities, the experimental endpoints were considered when tumour volume reached 1000 mm^3^ as commonly reported for subcutaneous tumours. Both the relative tumour volume, body weight change and the corresponding survival curves (with regard to the percent mice with tumour volumes < 1000 mm^3^) have been studied based on this advanced model, as shown in Fig. 7. At day 15 and day 19, the volumes of mice treated with PBS and NGs exceeded the defined end-point 1000 mm^3^, respectively, with the P value of 0.0053 and 0.0065 showing significant differences comparing with the tumor volumes of SCNGs group (Fig. 7a). At day 23, all of the mice volume treated with PBS and NGs exceeded 1000 mm^3^, whereas 83% of SCNGs-treated mice volume remained lower than the end-point size. The administration of SCNGs significantly prolonged the survival of the mice, as shown in Fig. 7c. Concurrently, a more detailed histopathology analysis of the HCC PDX tumour tissue after different treatments has been conducted to verify the actual therapeutic species (Fig. 7d). The localization of SCNGs in tumour tissues was confirmed by ex vivo Prussian blue staining images of tumour tissue extracted from the mice after treatment. We can clearly see that parts of the tumour tissues were stained blue marked by the arrows, indicative of the accumulation of iron oxide-based SCNGs within the tumour areas. For a more quantitative data, the ICP test has been also conducted with about 32.6 ng Fe per mg tumour tissue. The further EDT related therapeutic species including the ROS, ^1^O_2_ and the intermediate HClO have been detected in tumour tissues of different groups by using the corresponding fluorescent probes, DCFH-DA, SOSG and aminophenyl fluorescein (APF, as HClO detector). Based on the obtained results, the strong green fluorescence of DCFH-DA and SOSG can be detected on the slice of SCNGs group, comparing to the PBS and NGs groups. Only SCNGs can effectively upregulate the level of ROS and ^1^O_2_ to fulfil the responsive EDT, similarly to the in vitro results; finally, the pathological examinations of related tumour tissues by H&E staining also show the consistent results with the previous data on HepG2 nude mice model. Significant damaged and apoptotic tumour cells can be found in the observed tumour tissues, while negligible damage in the organ tissues can be detected (Supplementary Fig. 9) of SCNGs group.

## Discussion

In this study, bioinspired by the mechanism of neutrophil lysosomes, an EDT protocol has been proposed, which employs the endogenous-like enzymes as the core component to cascade catalyze ROS in tumour microenvironment to drastically produce ^1^O_2_. The supramolecular hybrid nanogel via the developed facile AP-triggered self-assembly with cascade enzyme system of SOD and CPO has been constructed (SCNGs). The supramolecular hydrogel matrix here can relieve the enzyme protein degradation by the non-covalent immobilization and sacrifice protection by their protein-like structure, ensuring high structural stability and enzymatic capability for efficient catalysis; the tandem SOD and CPO in the hybrid nanogels endow the SCNGs with excellent ROS sensitivity and capability of significantly promoted oxidative stress of tumour cells. The encapsulated CPO can convert H_2_O_2_ into ^1^O_2_ in tumour cells, while SOD can consume the endogenous ˙O_2_ˉ to increase the endogenous H_2_O_2_ levels either to feed CPO or to kill tumour cells. In vitro results of EDT on HepG2 cells have shown that the intracellular ROS activity can be greatly enhanced by the cascade reaction of SOD and CPO in SCNG inducing considerable cytotoxicity. While the endogenous ROS in HL-7702 cells cannot be catalysed to trigger much enough killing ROS to induce the cell apoptosis, due to the strong antioxidant response in normal cells comparing to the tumour cells. In vivo experiments on both HepG2 cell derived nude mice model and the advanced HCC PDX mice model, further demonstrate that the tumour microenvironment-responsive SCNGs can significantly inhibit proliferation and enhance apoptosis of the tumour cells/tissues. The further histopathology analysis by using the corresponding fluorescent probes, DCFH-DA, SOSG and APF indicates that SCNGs can effectively upregulate the level of ROS and ^1^O_2_ species to fulfil the responsive and efficient EDT, similarly to the in vitro results.

In summary, as a proof of concept study, a simulation of neutrophil lysosomes has been constructed by AP-triggered self-assembled core-shell nanogel, co-loading with cascade SOD and CPO to responsively and controllablely generate ^1^O_2_ quickly killing abnormal cells, as a proposed EDT. This multifunctional SOD/CPO-loaded nanogel system, SCNG can responsively generate the therapeutic ^1^O_2_ around the simulated ROS microenvironment and in the tumour cells with high efficacy. The SCNGs show good aqueous dispersibility, considerable biocompatibility, and negligible toxic effects to normal cells and (conversely) strong cytotoxicity to cancer cells, which can be further served as safe and efficient potential therapeutic agents for EDT of tumours. We believe that this work will provide a insight to establish non-invasive and endogenous therapeutic nanoplatform only relying on the endogenous ROS in tumour without any other external intervention. The potential and unique efficacy and safety advantages due to its highly enzymatic efficiency and specificity can serve EDT as a very favorable constituent part in ^1^O_2_-induced tumour therapeutic strategy, such as PDT, SDT and CDT.

## Methods

### Materials

Ferric chloride hexahydrate (FeCl_3_·6H_2_O), ethylene glycol (EG), diethylene glycol (DEG), Acid Phosphatase (AP, M_W_ = 10 kDa, EC 3.1.3.2), Superoxide Dismutase (SOD, ≥ 3000 U mg^−1^, EC 1.15.1.1) and Chloroperoxidase (CPO, ≥ 3000 U mL^−1^, EC 1.11.1.10) were purchased from Sigma-Aldrich. Sodium acetate (CH_3_COONa, NaOAc), Poly (vinylpyrrolidone) (PVP, K30), Pyrogallol, Succinic anhydride and 3-aminopropyltriethoxysilane (APTES) was purchased from Aladdin. 1-ethyl-3-(3-dimethylaminopropyl) carbodiimide (EDC) and N-hydroxysuccinimide (NHS) were purchased from Energy Chemical, Fmoc-Tyr(H_2_PO_3_)-OH was purchased from GL Biochem. Tris·HCl buffer was purchased from Beijing Solarbio Science & Technology. Singlet Oxygen Sensor Green (SOSG) was purchased from Thermofisher. The aminophenyl fluorescein (APF) as the HClO detector were purchased from Shanghai Maokangbio. Enhanced BCA Protein Assay Kit was purchased from Beyotime Biotechnology. All materials were used without further purification.

### Instruments

Scanning electron microscope (SEM) images were conducted on FEI Magellan 400 L system. TEM and STEM images were taken with a JEOL 2100 microscope (Japan) operated at 200 kV with element mapping (Oxford X-Max 80T). Dynamic light scattering (DLS) studies of the microgels were conducted on Zetasizer Nano instrument (Malvern Instruments Ltd., United Kingdom). UV-vis spectra were obtained by UV-2700 (Shimadzu Corporation). Absorbance of the CCK8 assay was measured at 450 nm using the ELx800 reader (BioTek Instruments, Inc, Winooski, VT). FACS was analyzed using the Becton–Dickinson spectrophotometer (BD, Franklin Lakes, NJ). Confocal microscopic studies were performed on a confocal fluorescence microscope Zeiss LSM510 and LSM710 system (Carl Zeiss AG, Jena, Germany). Thermogravimetric analysis (TG) of MNPs and nanogels were carried out on a thermal analyzer (NETZSCH STA 409 PC, Germany).

### Preparation of the SCNG nanoparticles

The monodispersed Fe_3_O_4_ (MNPs) were synthesized according to the literature to produce a diameter of 80 nm^39^. Afterwards, the obtained MNPs (1 mL, 10 mg mL^−1^) was first resuspended in a mixture of 80 mL of ethanol and 80 mL of deionized water and sonicated for 30 min. Then, 6 mL of APTES was injected and stirred at 70 °C for 24 h under continuous N_2_ flow for amination. The amino-functionalized MNPs (MNPs-NH_2_) were obtained after washing three times with ethanol and deionized water. Subsequently, the MNPs- NH_2_ was further treated with 10% succinic anhydride in dimethylformamide (DMF) and stirred for 18 h to achieve carboxylic functionalized nanoparticles (MNPs-COOH). The resultant mixture was purified by magnetic separation and washed with ethanol and deionized water for further use.

The acquired MNPs-COOH was then activated by EDC (200 mg) and NHS (300 mg) in 20 mL of phosphate buffer solution at pH 5.8 for 2 h. After 3 washing steps, the nanoparticles were resuspended in 20 mL of AP solution (40 mg, 2 mg mL^−1^) to covalently attach the AP on the surface of MNPs. After separation, the obtained MNPs@AP nanoparticles were further dispersed in 10 mL of precursor coating solution, which was composed of Fmoc-Tyr(H_2_PO_3_)-OH (0.5%) and Na_2_CO_3_ (0.2%), and mechanically stirred for 24 h at room temperature. The supramolecular NGs were then collected by magnetic separation after washing three times with deionized water. Finally, 0.75 mL of the mixture of SOD (0.6 mg, 2000 U) and CPO (70 μL, 1200 U) in Tris·HCl buffer (1 M, pH 6.0) were added into the NGs solution (25 mg, 20 mg mL^−1^) and stirred at room temperature for another 24 h. The dual enzyme-loaded NGs (SCNGs) were acquired by magnetic separation. The supernatant was analysed to calculate the amount of residual enzyme.

### Loading Amount and Activity Test of SOD and CPO

The loading amount and activity of SOD within SCNGs was determined based on the inhibition ability of SOD on pyrogallol autoxidation. The inhibition rate of pyrogallol autoxidation can be expressed as the following equation 1:

1 $${\mathrm{inhibition}}\left( \% \right) = \left( {{{A}} - {{B}}} \right) \times 100/{{A}},$$

where A and B represent the autoxidation rates of pyrogallol in the absence and presence of SOD, respectively. The autoxidation rate of pyrogallol can be calculated by the slope of absorbance curve during the process of autoxidation at the first minute. Therefore, the amount of residual SOD in supernatant can be measured by the comparison of its inhibition rate to the standard quantitative free SOD. Then, the amount of loading SOD can be quantified by the subtraction of initial added SOD and residual SOD in supernatant. Typically, 10 μL standard free SOD (0.1 mg mL^−1^) in PBS (pH 7.8, 50 mM) or 10 μL residual SOD in supernatant (diluted by 4 times) and 2.98 mL Tris·HCl buffer (50 mM, pH 8.2, including 1 mM Na_2_EDTA) were first added to a quartz cuvette, followed by injecting of 10 μL pyrogallol (50 mM in 10 mM HCl). Immediately, the kinetics measurements were performed by a UV spectroscopy with the absorbance peak at 325 nm and 6 s interval. The absorbance change at 325 nm was recorded to calculate the autoxidation rate of pyrogallol, and the amount of loading SOD can thus be quantified. The activity of the immobilized SOD in nanoparticles is defined as the ratio between the inhibition of sample and free SOD:

2 $${\mathrm{Activity}}_{{\mathrm{SOD}}}\left( \% \right) = {{I}}_{{\mathrm{confined}}} \times 100/{{I}}_{{\mathrm{free}}},$$

where *I*_confined_ and *I*_free_ represent the inhibition rates of pyrogallol autoxidation by SOD-laden NGs (SCNGs) and free SOD, respectively. The experiment recipe was the same as above, and the amount of SOD employed in the parallel experiment should be equivalent. The absorbance change at the first minute was assigned to compare the activity of SOD in different states, such as free SOD and SCNGs.

The loading amount of CPO was determined by the UV absorbance of the residual CPO in the supernatant at 403 nm. The activity of CPO was evaluated based on the ability of CPO to catalyze the conversion of monochlorodimedon (MCD) to dichlorodimedon (DCD) at pH 2.75 in the presence of potassium chloride (KCl) and H_2_O_2_. The activity assay was performed in 0.1 M phosphate buffer (pH 2.75), containing 20 mM KCl, 2 mM H_2_O_2_, 0.1 mM MCD and standard free CPO (2 U) or SCNGs (including 2 U CPO) in supernatant. The reaction progress was monitored by recording the absorbance changes at 278 nm. The decrement of MCD in the initial first minute was employed to evaluate the activity of CPO. After quantification of CPO in SCNGs, the activity comparison assays of CPO in different states (free CPO or SCNGs) ware further conducted, which were performed the same as above and the amount of CPO in different states should be equivalent. The reaction rate at the first minute was employed to evaluate the activity of free CPO and SCNGs. The specific activity of SCNGs was expressed as the reaction rate comparison of SCNGs relative to free CPO:

3 $${\mathrm{Activity}}_{{\mathrm{CPO}}}\left( {\mathrm{\% }} \right) = {{R}}_{{\mathrm{confined}}}{\mathrm{ \times }}100/{\mathrm{R}}_{{{free}}},$$

where *R*_confined_ and *R*_free_ represent the reaction rate of SCNGs and free CPO, respectively.

The activities of the SOD and CPO in SCNGs at different pH (pH = 4.6, 6.0, 7.0) environment were further tested according to the above methods. Moreover, the storage activities of SOD and CPO in SCNGs were also performed during 30 days at different time interval. The cumulative release of the enzymes in PBS at 37 ℃ were conducted by detecting the total amount of the protein at different time points during 14 days using the BCA protein quantitation kit.

### Detection of singlet oxygen in vitro

The EPR spectra were recorded on a Bruker EMX-8/2.7 spectrometer operating at 9.873 GHz (microwave power: 20 mW; modulation frequency: 100 kHz; modulation amplitude: 0.5 G; receiver gain: 4 × 10^5^). The mixture of NaCl (100 mM), H_2_O_2_ (100 μM) and SCNGs or free CPO (including 8 U CPO) with the ^1^O_2_ trapper (TEMP) was rapidly transferred to a standard quartz capillary and placed into the EPR spectrometer. The EPR spectrum was then recorded every 4 min. Another assay was performed as following: NaCl (100 mM), XO (0.26 U), X (10 mM) and free SOD (60 U), free CPO (40 U) or free SOD/CPO (including 60 U SOD and 40 U CPO) was rapidly mixed with the ^1^O_2_ trapper (TEMP) to monitor the EPR spectrometer immediately.

SOSG was used as the probe molecule to detect the generation efficiency of ^1^O_2_ in SCNGs. Typically, 10 μL of SOSG (50 μM) was added into the solution composed of NaCl (100 μL, 1 M), H_2_O_2_ (10 μL, 10 mM) and SCNGs or free SOD/CPO (including 10 U SOD and 8 U CPO) to form a 1 mL solution in the cuvette. The fluorescence intensity was then recorded on an F-7000 fluorescence spectrophotometer (Hitachi, Japan) at different time intervals. ^1^O_2_ detection was conducted by recording the fluorescence emission spectra of SOSG (Ex/Em = 504/525 nm) with the excitation wavelength fixed at 488 nm.

HepG2 and HL-7702 cells were obtained from the Institute of Biochemistry and Cell Biology, Shanghai Institutes of Biological Sciences, Chinese Academy of Sciences (Shanghai, China). All cells were tested negative for mycoplasma contamination. To study the production of ^1^O_2_ in cancer cells, the HepG2 cells were seeded in confocal dish with a density of 5 × 10^4^ per well. After incubation for 24 h, the medium was replaced with fresh culture medium. Then, PBS, NGs (300 µg mL^−1^) or SCNGs (300 µg mL^−1^) was injected to the well. After further incubation for 2 h, the cells were treated with SOSG probe (5 µM). Subsequently, the fluorescence emission spectrum of oxidized SOSG (E_x_/E_m_ = 504/525 nm) was immediately recorded on a confocal fluorescence microscope (Carl Zeiss AG, Jena, Germany). Measurements were made for 140-second periods for 20 pictures, with 40-second intervals between each measurement. 10 group of 3D-CLSM pictures were captured automatically using 30 min (3 min/group). For fully showing the living cell fluorescence intensity, the photomicrograph at 18 min with YZ and XZ planes were captured.

### In vitro cell cytotoxicity

For cell viability analysis, the HepG2 cells were seeded into 96-well plates with a density of 5000 cells per well for 24 h. And then, the cells were randomly treated with NGs and SCNGs at different concentrations from 1 μg mL^−1^ to 500 μg mL^−1^. After further incubation for 24 h, a mixed solution consisting of CCK-8 (10 µL, Dojindo, Kumamoto, Japan) and fresh culture medium (100 µL) was added to each well and incubated for an additional 2 h at 37 °C and 5% CO_2_. Finally, the absorbance at 450 nm was measured by a microplate reader (BioTek Instruments, Inc., USA), and the IC_50_ value was calculated using the SPSS statistics 21.0. Besides, to further evaluate the safety of SCNGs, the cytotoxicity test was also conducted by CCK8 assays with HL-7702 cells (the normal hepatic cell).

For cell apoptosis analysis, the HepG2 cells were firstly treated with NGs or SCNGs at the IC_50_ value for 24 h. And then, the cells were preincubated with 5 µL annexin V (5 µL annexin V-FITC was dissolved in 50 µL buffer, Bio Basic Inc., Markham, ON, Canada) in dark at room temperature for 15 min, followed by adding 10 µL PI (10 µL PI was dissolved in 250 µL buffer, Sigma). After staining, the percentage of apoptotic cells was analysed by flow cytometry (BD, Franklin Lakes, NJ). The Q2 region represents late apoptotic cells, and Q4 region represents early apoptotic cells. Typical gating used is shown in Supplementary Fig. 10.

### Analysis of oxidative stress in cells

The HepG2 and HL-7702 cells were seeded with a density of 1 × 10^5^ per well in 6-well plates, which treated with PBS, NGs or SCNGs at IC_50_ value for 24 h. Then, the cells were further loaded with 5-chloromethyl-2,7-dichlorodihydrofluorescein diacetate acetylester (DCFH-DA, 25 µM, Sigma-Aldrich) and the fluorescence emission spectrum of carboxy-DCF (Ex/Em = 495/529 nm) was immediately captured on confocal fluorescence microscope Zeiss LSM510 and LSM710 system (Carl Zeiss AG, Jena, Germany) at 0, 6, 12, 18, 30 min.

Quantitative analyses for ROS production were further performed by flow cytometric detection (BD, Franklin Lakes, NJ). The HepG2 cells after being treated with PBS, NGs or SCNGs (at IC_50_ value) were incubated with 25 µM DCFH-DA (Sigma-Aldrich) for 18 min. Then, the reaction of probe and ROS were stopped and cells were analysed by flow cytometry with excitation at 488 nm and emission at 505–550 nm.

### Immunofluorescence

The HepG2 cells were seeded with a density of 1 × 10^5^ per well in 6-well plates. After incubation for 24 h, the cells were randomly treated with PBS, NGs or SCNGs (at IC_50_ value) and further incubated for 24 h at 37 °C and 5% CO_2_. Then, the cells on the slides were fixed with 4% PFA at room temperature for 20 min and permeabilized with 0.5% Triton X-100 at 37 °C for 30 min, followed by incubation with anti-γH2AX primary antibody (Abcam, Anti-gamma H2A.X (phospho S139) antibody [9F3] (ab26350), dilution at 1:200) at 37 °C for 30 min. Then, the cells were stained and sealed with ProLong Gold Antifade Reagent plus 4′,6-diamidino-2-phenylindole (Invitrogen). Finally, the preparations were washed with PBS and mounted in fluorescent mounting medium with DAPI (Invitrogen). Negative controls were processed in the same way but without the primary antibody. Slides were photographed under a fluorescence microscope (Carl Zeiss AG, Jena, Germany).

### Analysis of DNA damage activity by comet assay

After treatment with PBS, NGs or SCNGs at IC_50_ value for 24 h, HepG2 cells were collected for analyzing DNA damage activity by comet assy. Cell density was adjusted to 1000 cells mixed with 0.5% low-melting point agarose equilibrated to 37 °C, and cell-agarose suspensions were spread onto the comet slides embedded with 1% normal-melting agarose for incubation at 4 °C. The prepared slides were lysed immediately in chilled neutral lysis buffer (0.1 M Na_2_EDTA·2H_2_O, 2.5 M NaCl, 1% Triton X-100, 10% DMSO, and 10 mM Tris, pH 8.0) at 4 °C in the dark for 4 h. In order to allow the cellular DNA to unwind, the slides were submerged in the precooled neutral electrophoresis buffer (90 mM Tris buffer, 90 mM boric acid, 2 mM Na_2_EDTA·2H_2_O, pH 8.5) at 4 °C for 20 min. Next, the damaged DNA fragments were electrophoresed for 30 min at a constant voltage of 20 V cm^−1^. After the slides being neutralized with 0.4 M Tris·HCl (pH 7.5) and left to air dry, samples were stained with the non-toxic DNA dye SYBR Green I at room temperature in the dark for 20 min and then examined under a fluorescence inverted microscope (DMI6000B; Leica, Wentzler, Germany).

### Cell cycle analysis

The HepG2 cells treated by PBS, NGs or SCNGs (at IC_50_ value) were suspended in ice-cold PBS and fixed in 70% ethanol at −20 °C for 18 h, after which the cells were washed with PBS and stained for 15 min at 37 °C with 500 μL of 50 μg mL^−1^ PI (containing 50 μg mL^−1^ RNase) (BD Pharmingen) followed by flow cytometric analysis (BD Franklin Lakes, NJ).

### Animal models

The animal experiments were conducted in accordance with the guidelines of the National Institutes of Health of China for the care and use of laboratory animals. All animal work was approved and conducted under the guidelines of Shanghai Tenth People’s Hospital, Tongji University’s Animal Care and Use Committee.

### HepG2 cell derived xenograft mouse model

Female nude mice (6–8 weeks) were purchased from Slaccas Co. Ltd. To establish the xenograft tumour model, the nude mice were subcutaneously injected with a suspension of 1 × 10^6^ HepG2 cells in PBS (80 mL) on the right hind limbs. When the tumour sizes reached about average size of 80–100 mm^3^, the mice were randomly divided into 3 groups: (1) PBS (100 μL) with intravenous injection; (2) NGs (100 μL, 5 mg mL^−1^, with the dose of 20 mg kg^−1^ mouse) with intravenous injection; (3) SCNGs (100 μL, 5 mg mL^−1^, with the dose of 20 mg kg^−1^ mouse) with intravenous injection. The mice were treated by intravenous injection at the 0 day, 2nd day, 4th day, 6th day and 10th day. The body weight was recorded, and tumor volume was measured by a calliper every two days which was calculated according to the following equation 4:

4 $${\mathrm{tumour}}\,{\mathrm{volume}}\left( {{\mathrm{mm}}^{\mathrm{3}}} \right){\mathrm{ = length \times width}}^{\mathrm{2}}{\mathrm{/2}}{\mathrm{.}}$$

### Hepatocellular carcinoma (HCC) patient derived xenograft (PDX) mouse model

HCC PDX model (No. 73) was ordered from the Shanghai Biomodel Organism by transferring patient tumour fragments to Male NOD-SCID mice (6–8 weeks). When the tumour sizes reached about average size of 80–100 mm^3^, the mice were randomly divided into 3 groups: (1) 6 mice with intravenous injection of PBS (100 μL); (2) 6 mice with intravenous injection of NGs (100 μL, 5 mg mL^−1^, with the dose of 20 mg kg^−1^ mouse); (3) 6 mice with intravenous injection of SCNGs (100 μL, 5 mg mL^−1^, with the dose of 20 mg kg^−1^ mouse).

The in vivo experiments have three investigators, who independently participated the whole experiments during the experimental period. As a special PDX model of individual differences, the experimental end point^50^ has been set when the tumour volume > 1000 mm^3^. The corresponding survival curves have been obtained based on the percent mice with tumour volumes > 1000 mm^3^. The plots in the illustrations of the corresponding tumour volumes and the body weights are terminated when the first animal in the group reaches a tumor volume of 1,000 mm^3^. Under the ethical approval conditions, the observation of mice have been prolonged to 31 days, until the size of once mouse tumors was evaluated to exceed 10% of the animal’s weight, all the experiments have been terminated as the completion of this experimental period. The mice were treated by intravenous injection at the 1st day, 2nd day, 4th day, 8th day, 12th day, 16th day, 20th day, 25th day and 30th day during the whole experimental duration. The body weight was recorded, and tumor volume was measured by a calliper every two days which was calculated according to the following equation 5:

5 $${\mathrm{tumour}}\;{\mathrm{volume}}\left( {{\mathrm{mm}}^{\mathrm{3}}} \right){\mathrm{ = length \times width}}^{\mathrm{2}}{\mathrm{/2}}{\mathrm{.}}$$

For further histological analysis, Prussian blue staining and ICP analysis, the mice were sacrificed, and the tumour tissues and major organs including livers, lungs, hearts, spleens and kidneys were collected. In addition, 6 mice were randomly chosen for intratumoural injecting PBS, NGs or SCNGs with SOSG or APF respectively to evaluate the ^1^O_2_ and HClO in tumour tissues.

### Histopathology analysis

The tumour tissues, livers, lungs, hearts, spleens and kidneys were excised and fixed in 10% neutral formaldehyde, conventionally paraffin embedded, sectioned, and placed on slides. For analysis, 4 μm sections from each sample were stained with haematoxylin and eosin (H&E, Sigma-Aldrich) for the histopathological evaluation using a standard procedure. The 4 μm tumour sections from intravenous injection groups were further immunohistochemically stained for TUNEL (terminal transferase UTP nick-end labelling) and KI-67 (nuclear-associated antigen KI-67, Ki-67 (D3B5) Rabbit mAb (Mouse Preferred; IHC Formulated) #12202, dilution at 1:400) assay to analyse the cell death and proliferation in tumour tissue. For ROS detection, the tumours from the three groups of PDX model mice were cryosectioned at 4 μm thickness and then stained with DCFH-DA according to the instructions. As for the detection of ^1^O_2_ and HOCl in vivo, the tumours from intratumoural injection of PBS, NGs or SCNGs with SOSG or APF on PDX model mice were collected and cryosectioned onto slides with a 4 μm thickness. Each tissue section was observed by a light microscopy or fluorescence microscope (Leica DMI6000). Sections were evaluated from six randomly selected fields by two separate pathologists in a blinded-manner.

### Statistical analysis

All data were expressed in this manuscript as mean ± s.d. All the results have been performed at least three times by independent experiments. No samples and animals were excluded from the analysis. A two-tailed Student’s *t* test was used to analyse the statistical significance between two groups. The statistical analysis was performed by using GraphPad prism 7.0 (GraphPad Software Inc.). Asterisks indicate significant differences (*P < 0.05, **P < 0.01).

## Supplementary information

Source Data

Supplementary Information

## Data Availability

A reporting summary for this Article is available as a Supplementary Information file. Additional data related to this paper may be requested from the corresponding authors (Wang Q. (wangqg66@tongji.edu.cn), Li J. (lijiyu@tongji.edu.cn) or Wang X. (15174@tongji.edu.cn)).
