# Supplementary material for: Cascade enzymes within self-assembled hybrid nanogel mimicked neutrophil lysosomes for singlet oxygen elevated cancer therapy
Source: Nat Commun. 2019 Jan 16;10:240. doi: 10.1038/s41467-018-08234-2 (PMC6335431; doi:10.1038/s41467-018-08234-2)
Supplement: Supplementary file 1 — Source Data [file 41467_2018_8234_MOESM1_ESM.zip › source data/Source Data-Figure 4/Source Data-Figure 4c,d.pptx]

## Slide 1
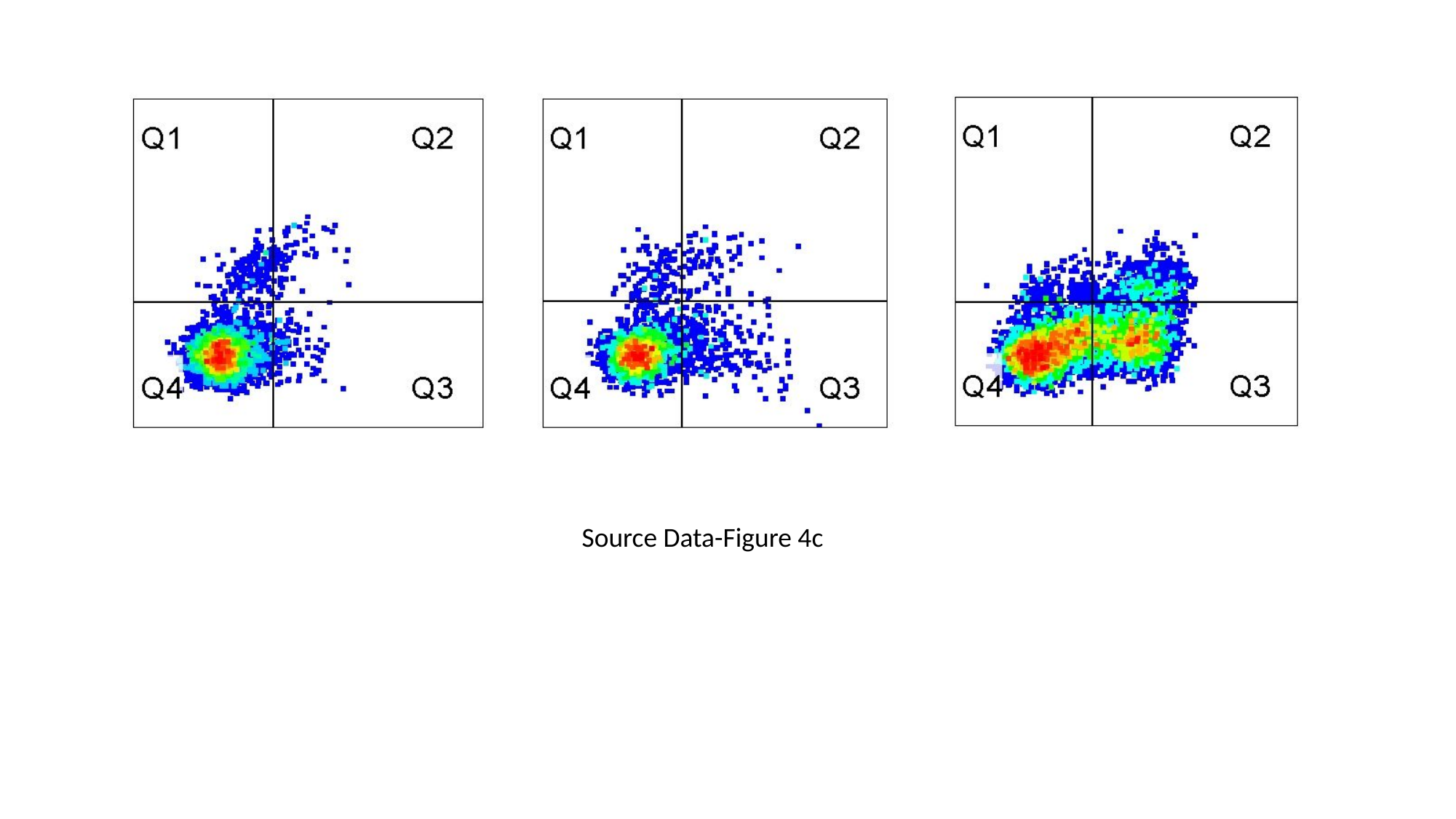

#
Source Data-Figure 4c

## Slide 2
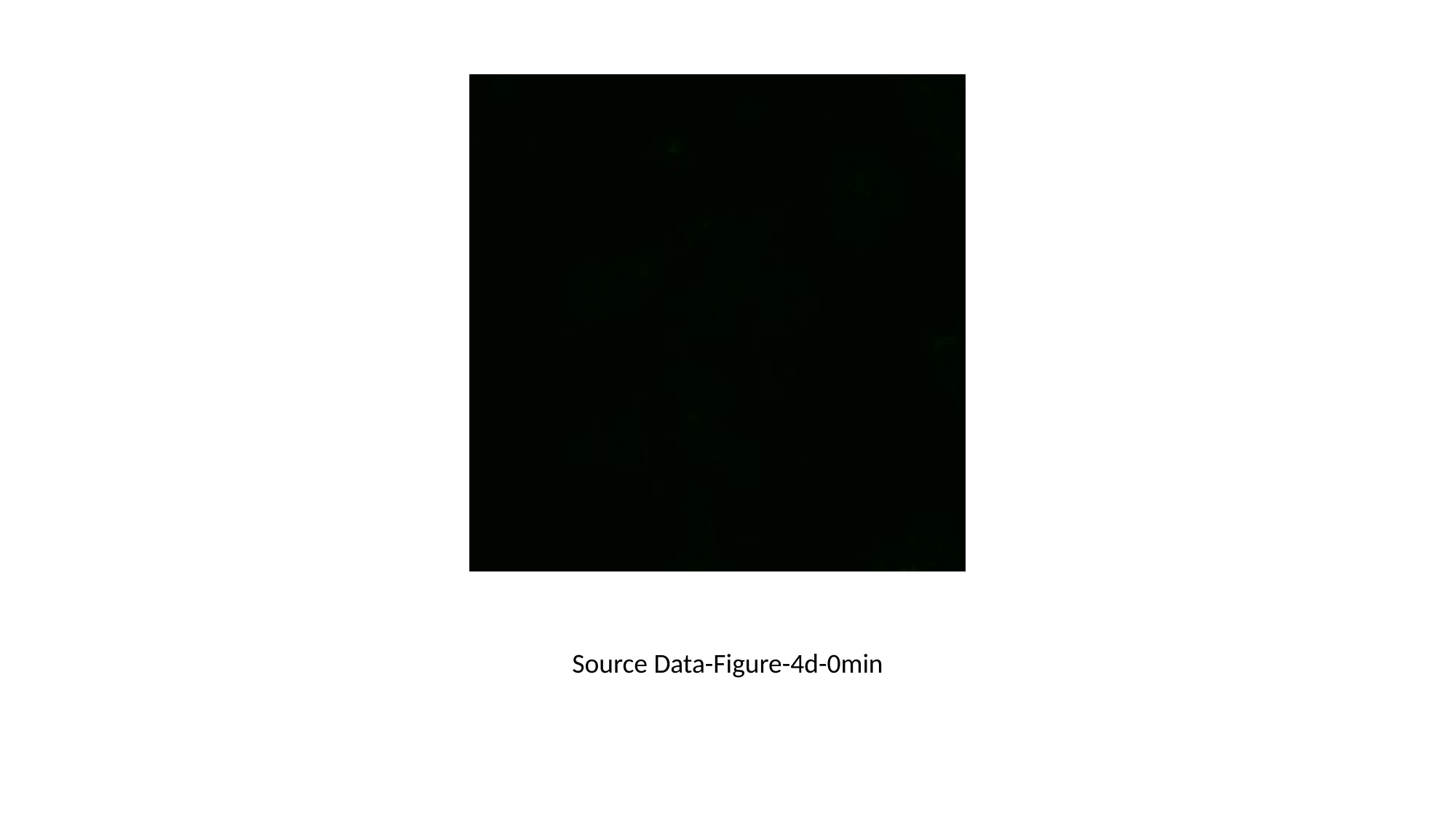

#
Source Data-Figure-4d-0min

## Slide 3
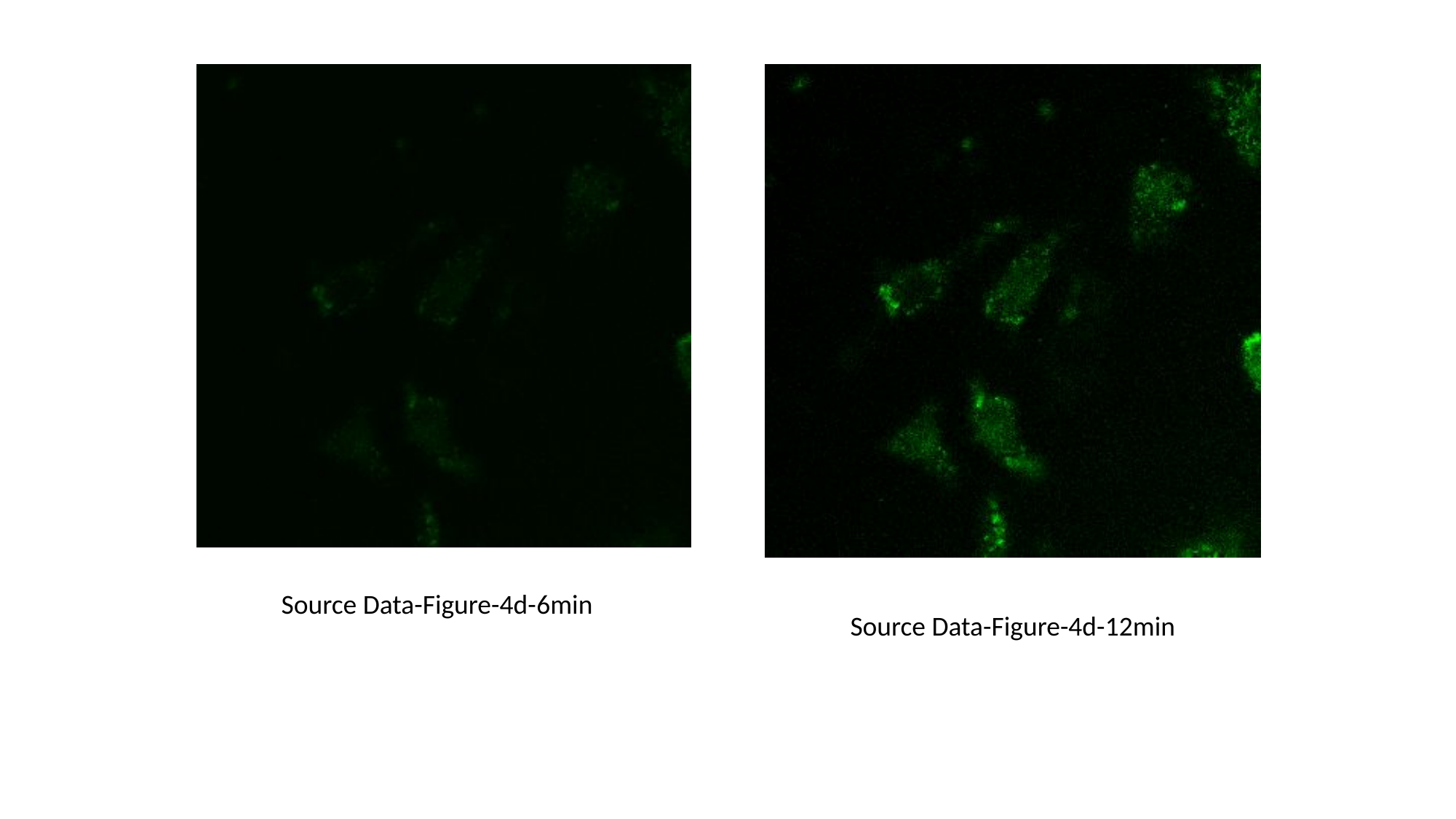

#
Source Data-Figure-4d-6min
Source Data-Figure-4d-12min

## Slide 4
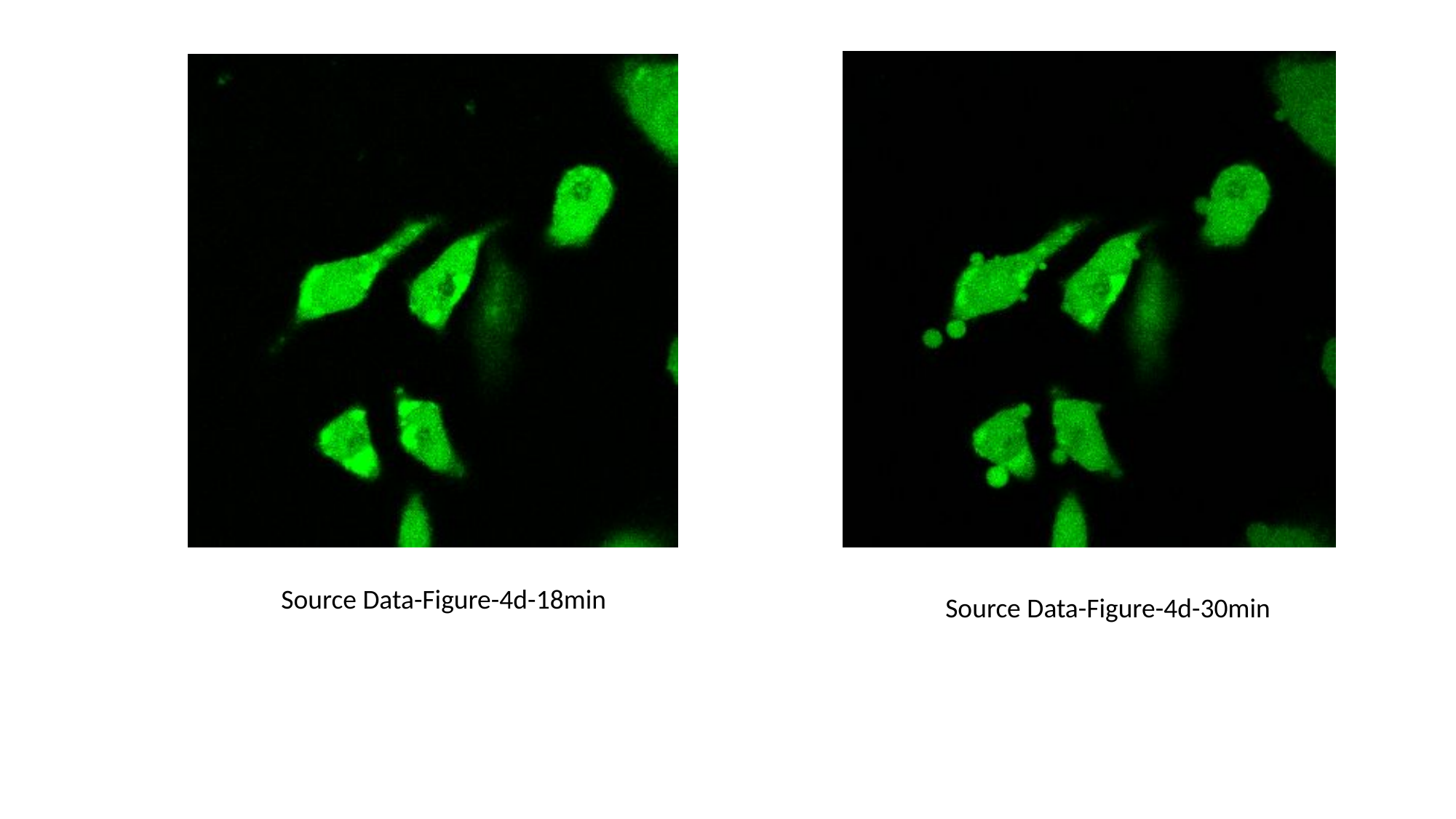

#
Source Data-Figure-4d-18min
Source Data-Figure-4d-30min

## Slide 5
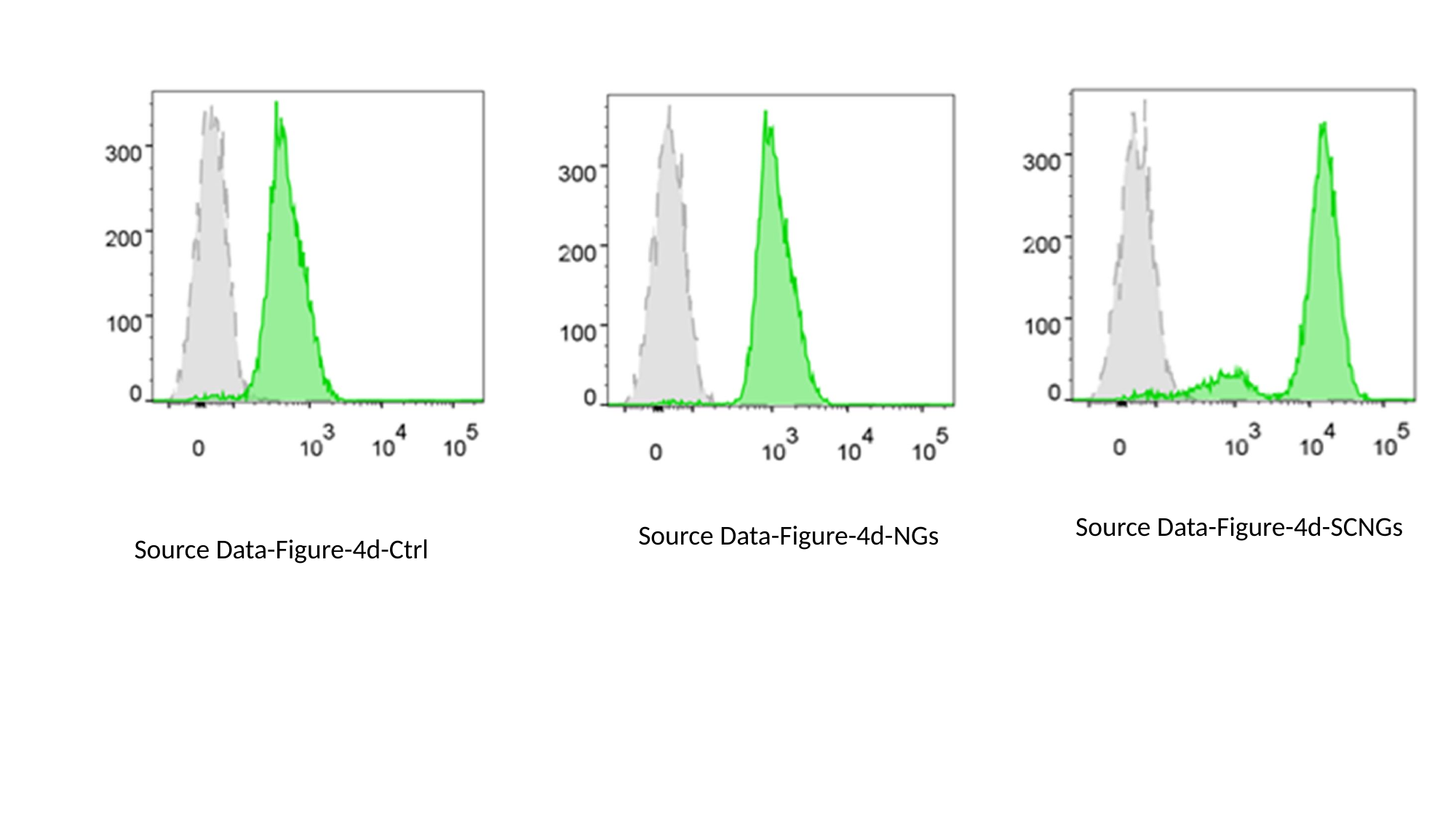

#
Source Data-Figure-4d-SCNGs
Source Data-Figure-4d-NGs
Source Data-Figure-4d-Ctrl
