# Supplementary material for: Cascade enzymes within self-assembled hybrid nanogel mimicked neutrophil lysosomes for singlet oxygen elevated cancer therapy
Source: Nat Commun. 2019 Jan 16;10:240. doi: 10.1038/s41467-018-08234-2 (PMC6335431; doi:10.1038/s41467-018-08234-2)
Supplement: Supplementary file 1 — Source Data [file 41467_2018_8234_MOESM1_ESM.zip › source data/Source Data-Figure 6/Source Data-Figure 6d-e.pptx]

## Slide 1
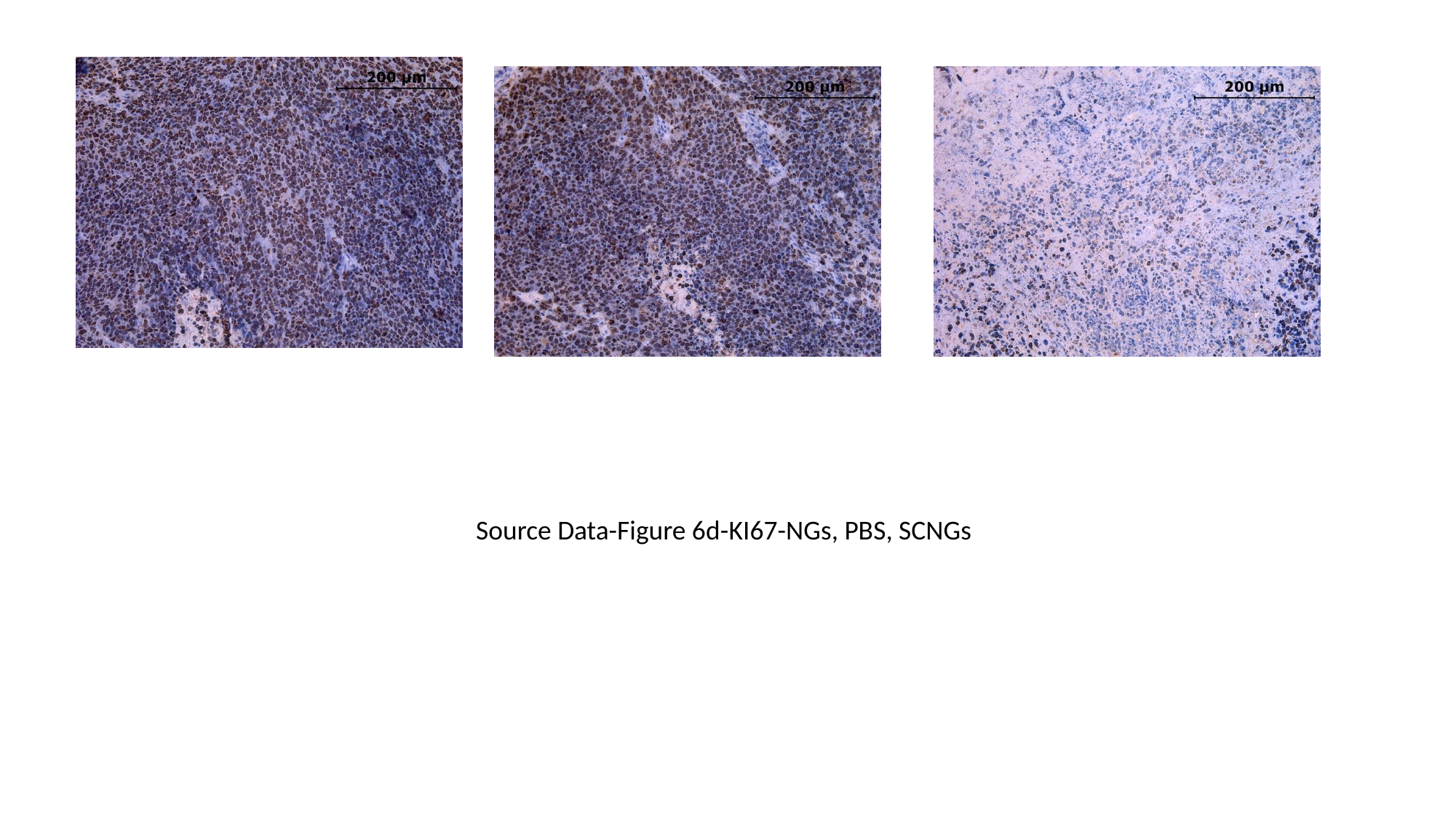

#
Source Data-Figure 6d-KI67-NGs, PBS, SCNGs

## Slide 2
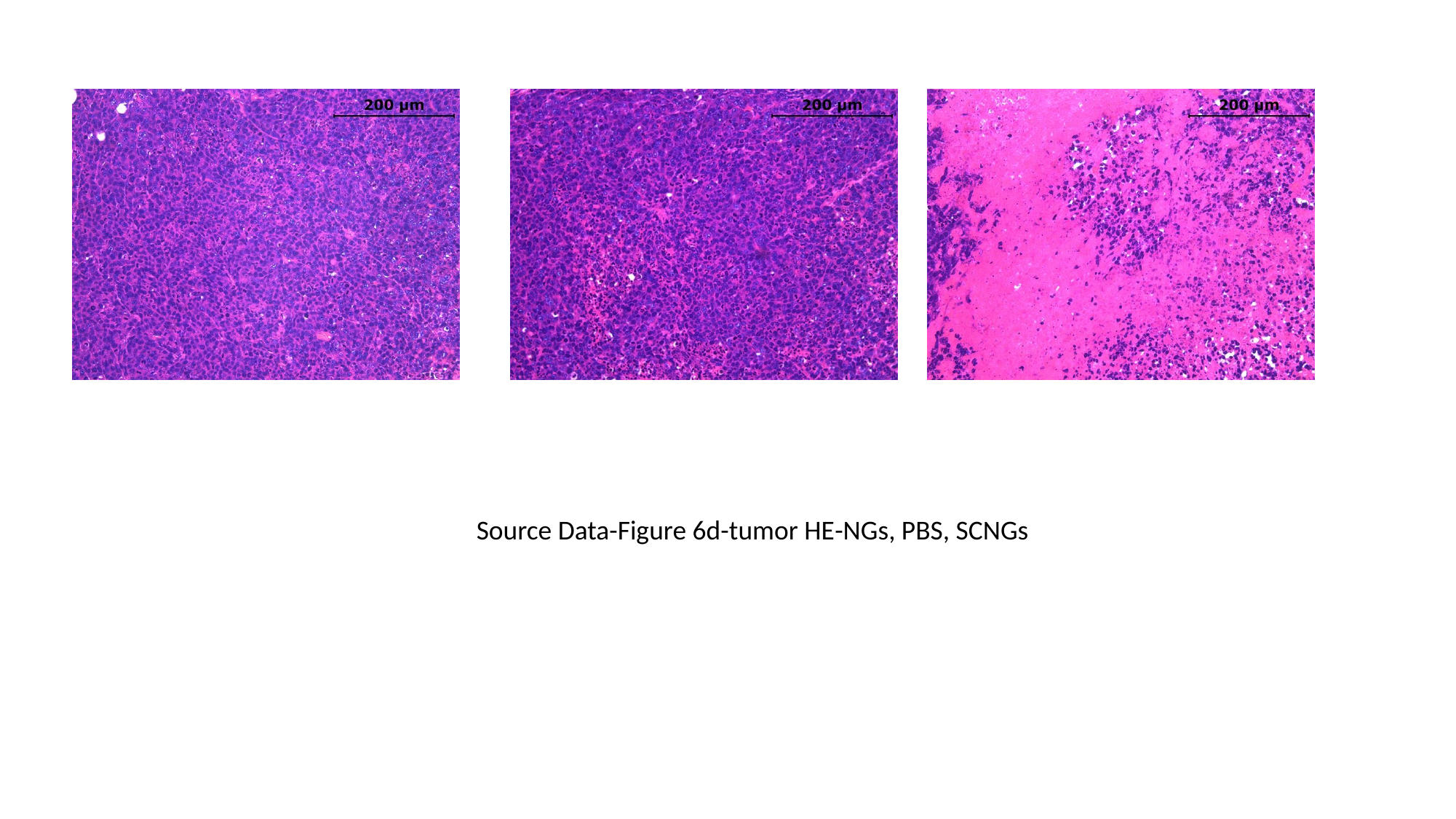

#
Source Data-Figure 6d-tumor HE-NGs, PBS, SCNGs

## Slide 3
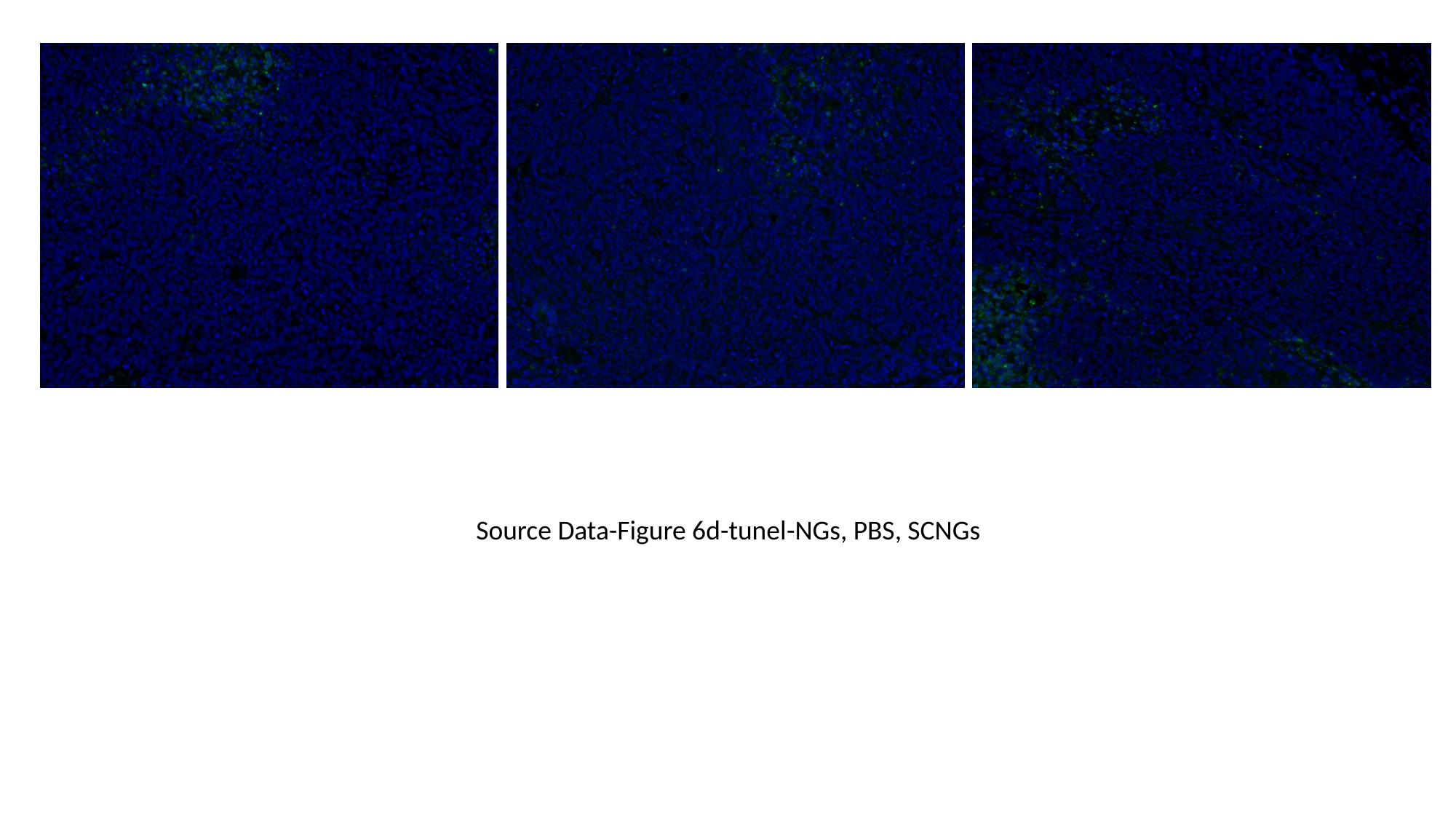

#
Source Data-Figure 6d-tunel-NGs, PBS, SCNGs

## Slide 4
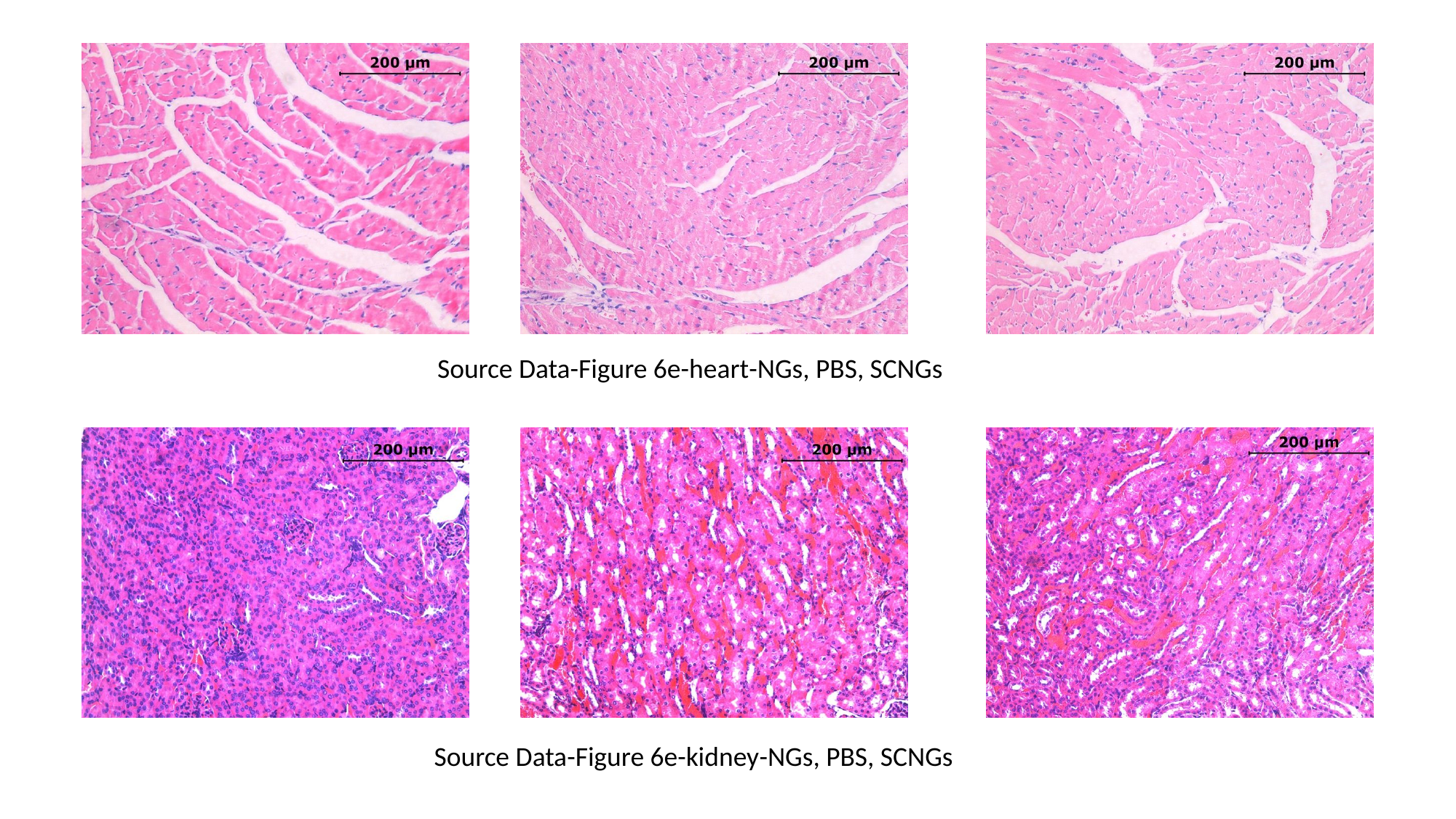

#
Source Data-Figure 6e-heart-NGs, PBS, SCNGs
Source Data-Figure 6e-kidney-NGs, PBS, SCNGs

## Slide 5
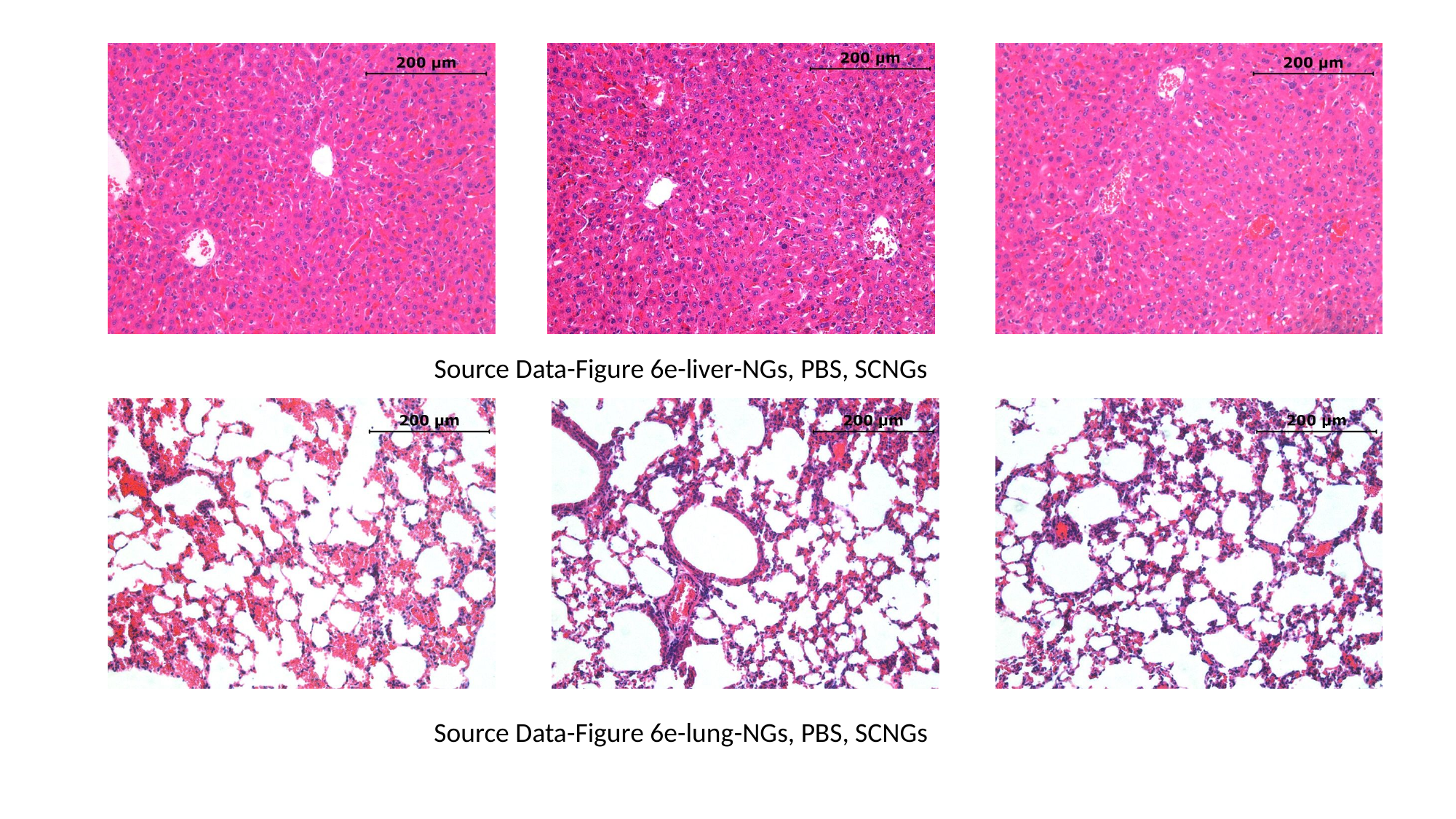

#
Source Data-Figure 6e-liver-NGs, PBS, SCNGs
Source Data-Figure 6e-lung-NGs, PBS, SCNGs

## Slide 6
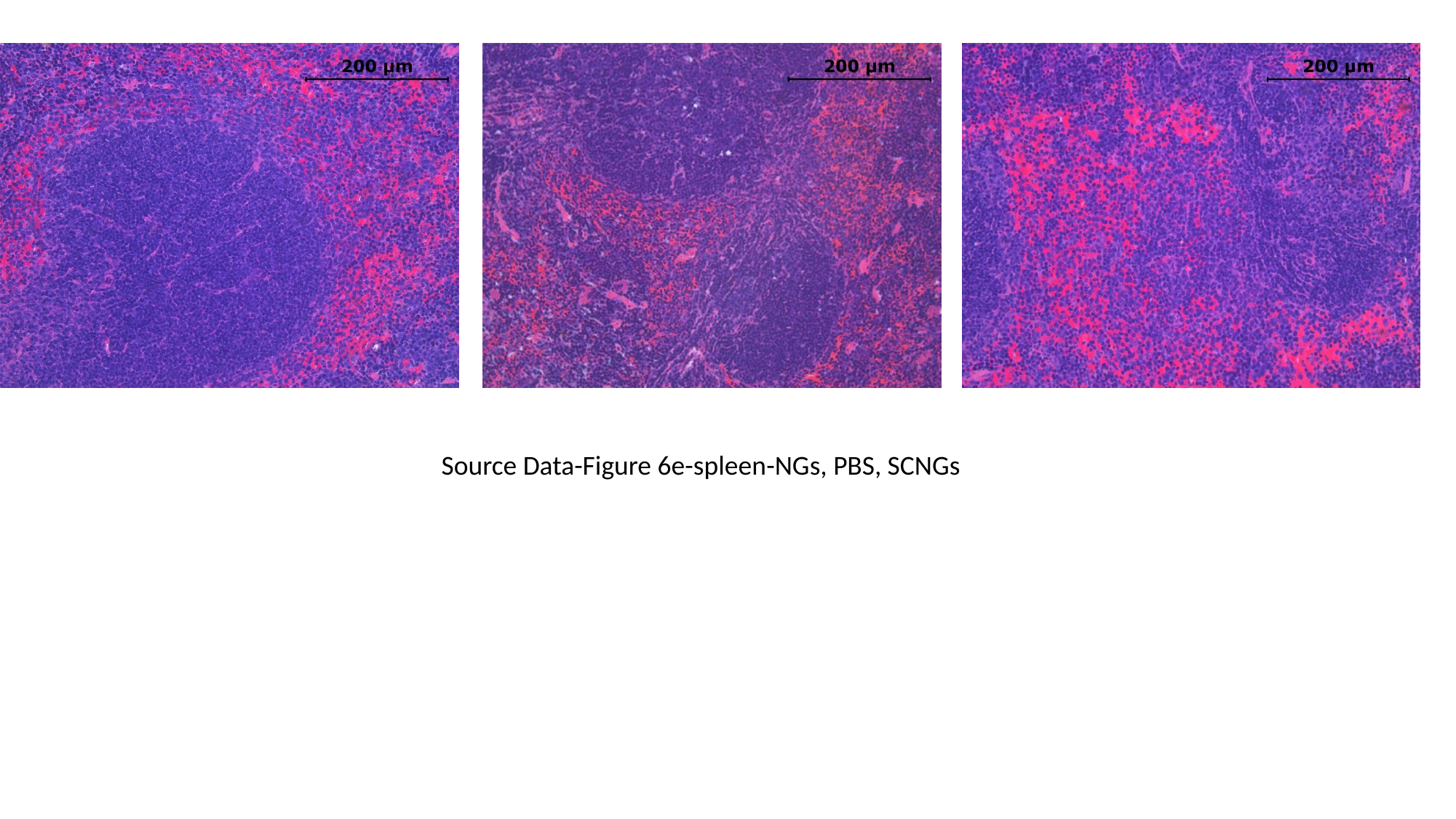

#
Source Data-Figure 6e-spleen-NGs, PBS, SCNGs
