# Supplementary figures and images for: Cascade enzymes within self-assembled hybrid nanogel mimicked neutrophil lysosomes for singlet oxygen elevated cancer therapy
Source: Nat Commun. 2019 Jan 16;10:240. doi: 10.1038/s41467-018-08234-2 (PMC6335431; doi:10.1038/s41467-018-08234-2)

## Slide 1
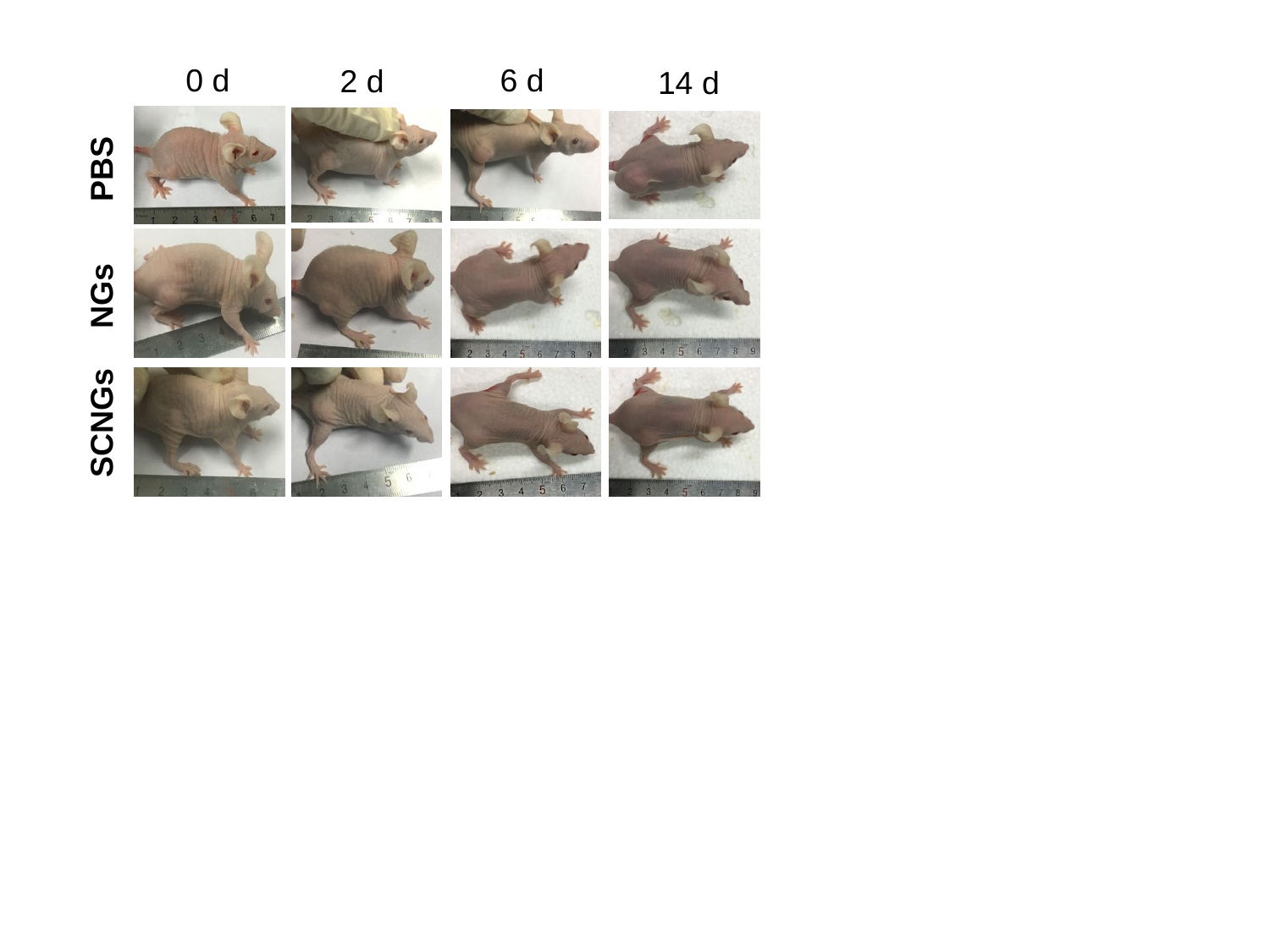

6 d
0 d
2 d
14 d
PBS
NGs
SCNGs

Supplement: Supplementary file 1 — Source Data [file 41467_2018_8234_MOESM1_ESM.zip › source data/Source Data-Figure 6/Source Data-Figure 6a.pptx]

## Slide 1
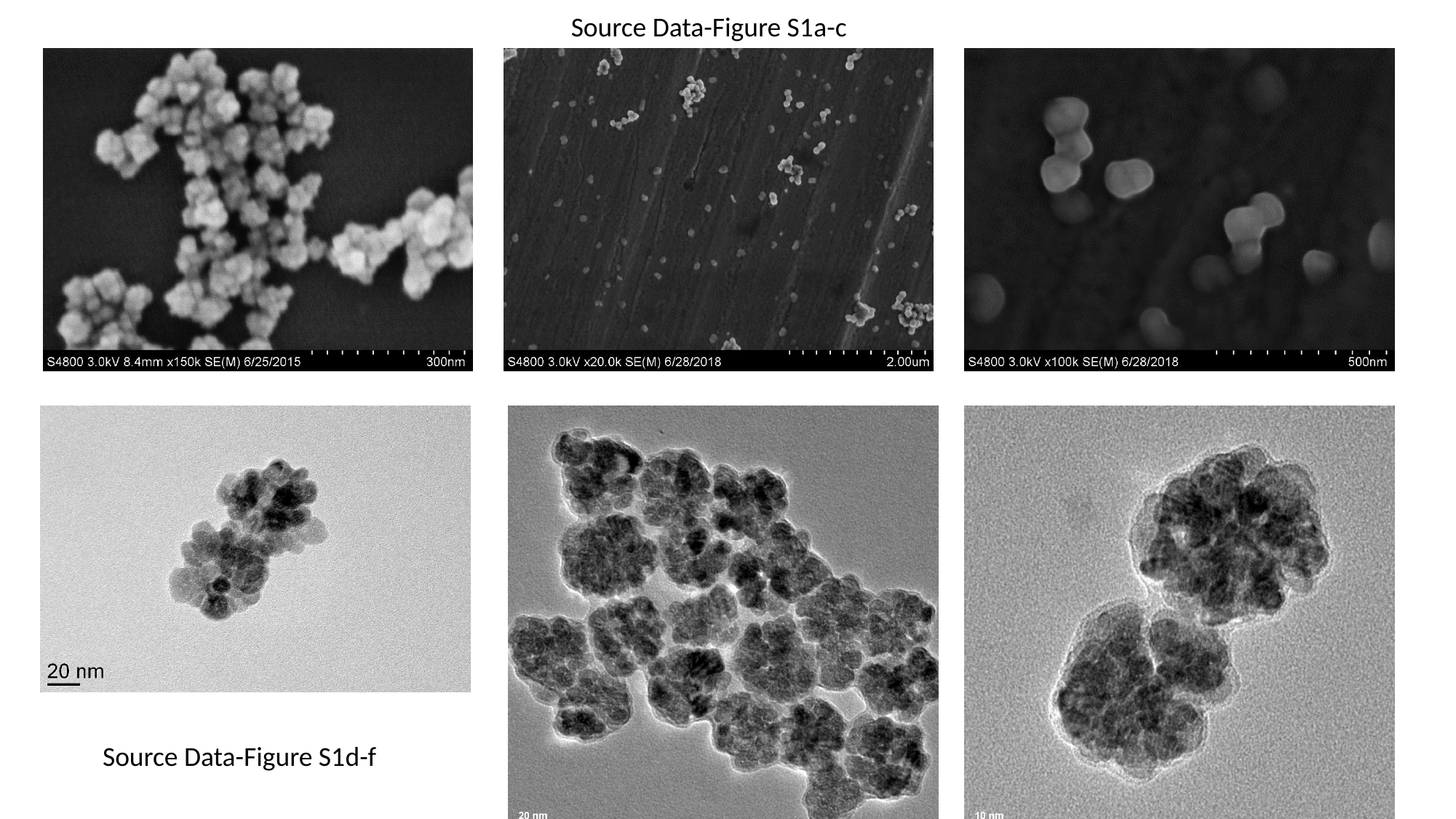

Source Data-Figure S1a-c
#
Source Data-Figure S1d-f

Supplement: Supplementary file 1 — Source Data [file 41467_2018_8234_MOESM1_ESM.zip › source data/Source Data-Figure S1/Source Data-Figure S1.pptx]

## Slide 1
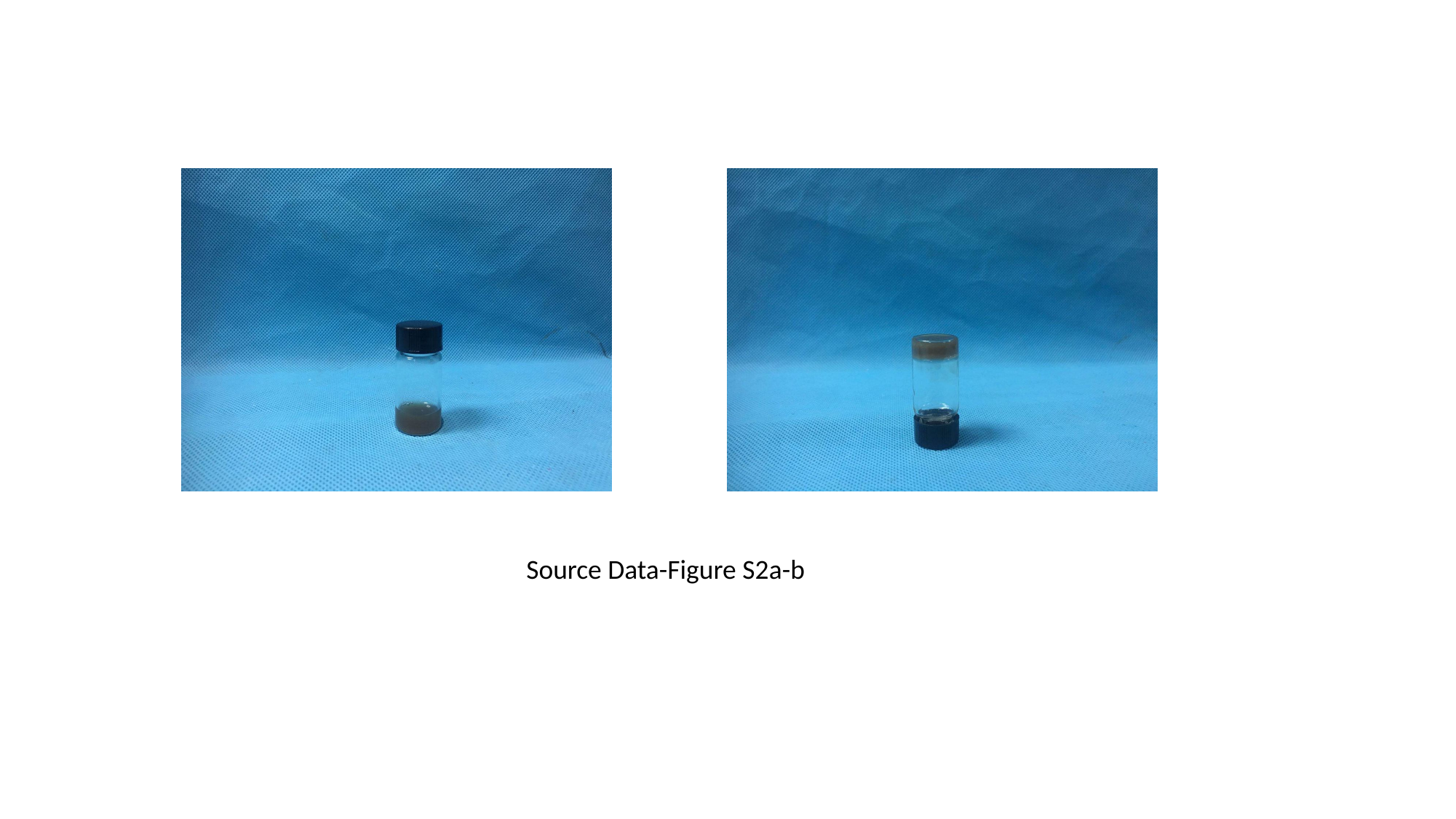

#
Source Data-Figure S2a-b

Supplement: Supplementary file 1 — Source Data [file 41467_2018_8234_MOESM1_ESM.zip › source data/Source Data-Figure S2/Source Data-Figure S2a-b.pptx]

## Slide 1
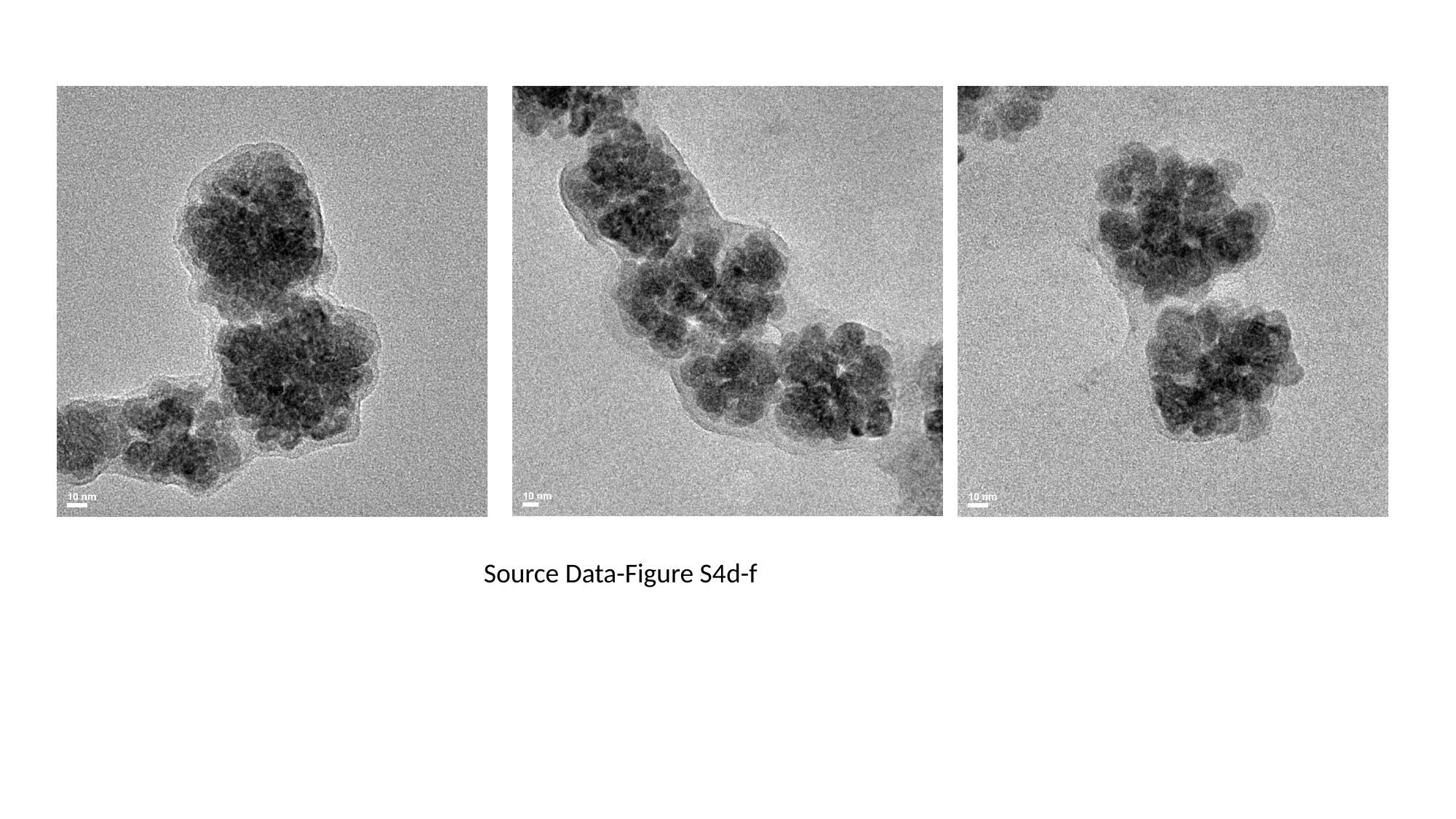

#
Source Data-Figure S4d-f

Supplement: Supplementary file 1 — Source Data [file 41467_2018_8234_MOESM1_ESM.zip › source data/Source Data-Figure S4/Source Data-Figure S4d-f.pptx]

## Slide 1
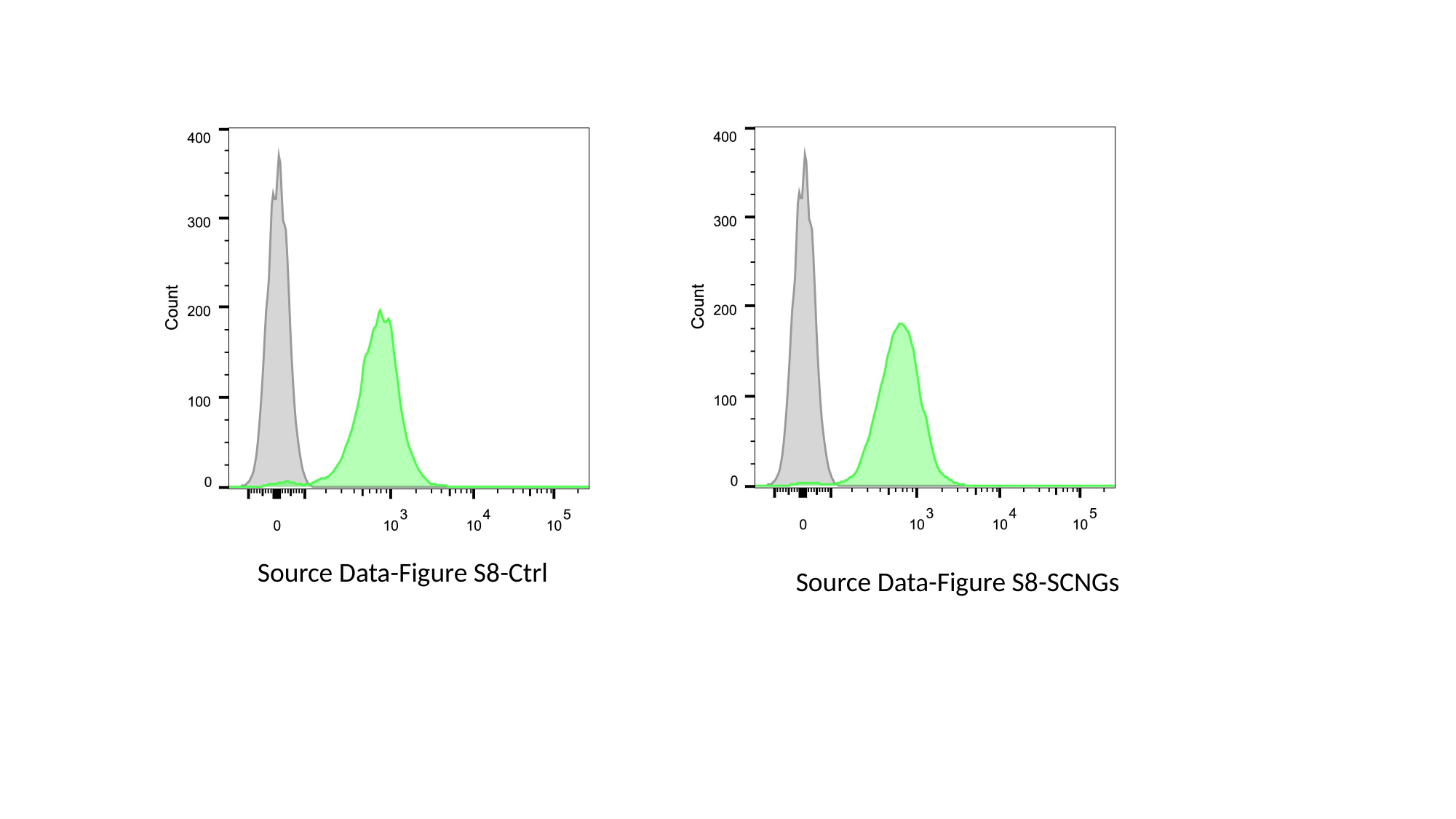

#
Source Data-Figure S8-Ctrl
Source Data-Figure S8-SCNGs

Supplement: Supplementary file 1 — Source Data [file 41467_2018_8234_MOESM1_ESM.zip › source data/Source Data-Figure S8/Source Data-Figure S8.pptx]
