# Supplementary material for: Cascade enzymes within self-assembled hybrid nanogel mimicked neutrophil lysosomes for singlet oxygen elevated cancer therapy
Source: Nat Commun. 2019 Jan 16;10:240. doi: 10.1038/s41467-018-08234-2 (PMC6335431; doi:10.1038/s41467-018-08234-2)
Supplement: Supplementary file 2 — Supplementary Information [file 41467_2018_8234_MOESM2_ESM.pdf]

Supporting Information for

Cascade Enzymes within Self-assembled Hybrid Nanogel Mimicked Neutrophil Lysosomes for Singlet Oxygen Elevating Cancer Therapy

Wu et al.

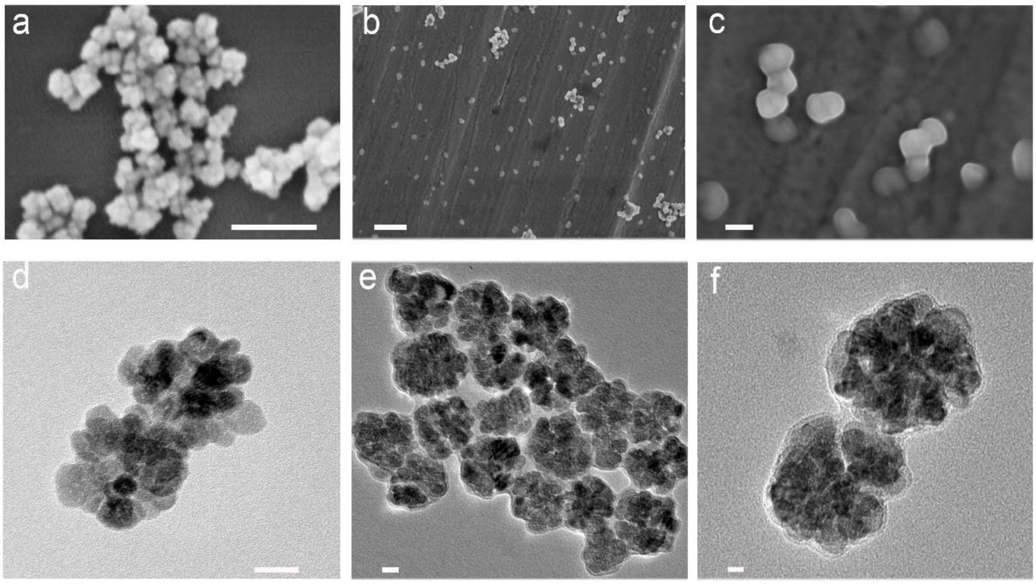

Supplementary Figure 1. SEM images of the MNPs (**a**) and SCNGs (**b**, **c**) and TEM images of the MNPs (**d**) and SCNGs (**e**, **f**). Scale bar, **a**, 200 nm. **b**, 1 μm. **c**, 100 nm. **d**, 20 nm. **e**, 20 nm. **f**, 10 nm.

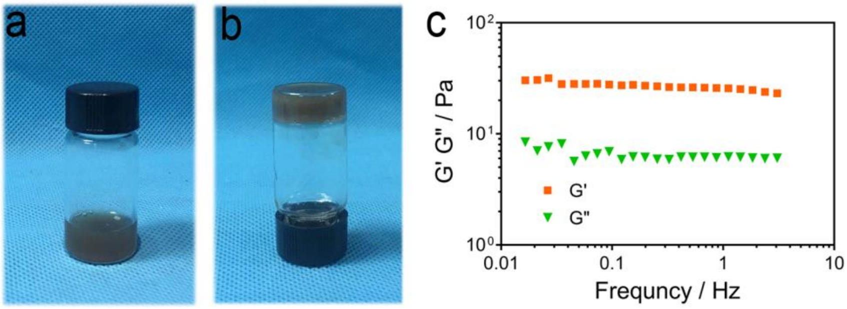

Supplementary Figure 2. The formation of the corresponding macro-hydrogel after adjusting the hydrogelators (**a-b**) and the corresponding frequency sweep tests of as-obtained macro-hydrogel (**c**).

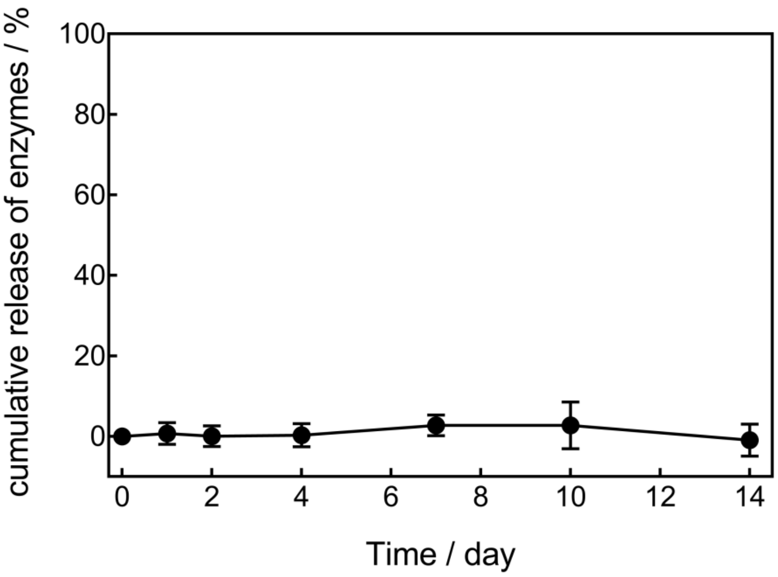

Supplementary Figure 3. The cumulative release of the enzymes in SCNGs in PBS at 37 ℃ for 14 days. The experiment was conducted three times, and representative results are present.

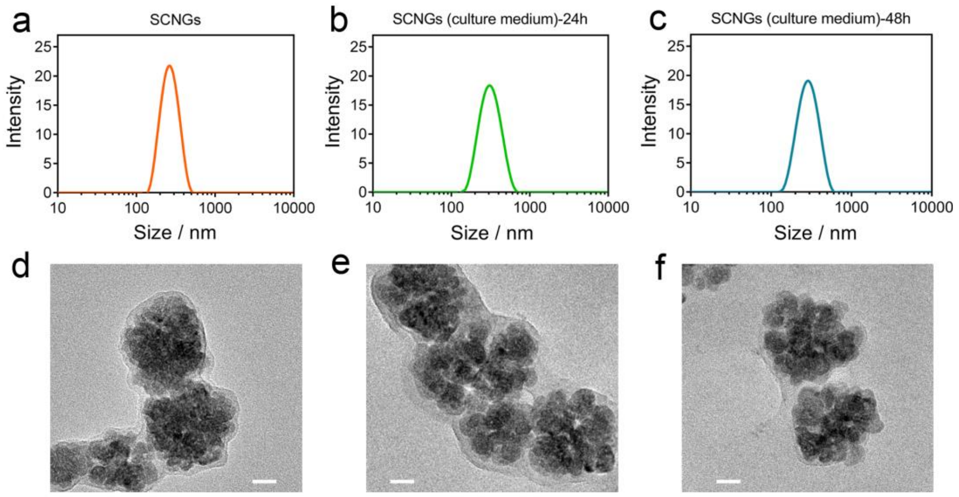

Supplementary Figure 4. The diameter distributions by DLS measurement and TEM imaging of SCNGs (**a, d**), SCNGs-24h (**b, e**) and SCNGs-48h (**c, f**) after co-incubation in the cell culture media containing 10% (v/v) fetal calf serum (FCS). Scale bar in **d**-**f**, 20 nm.

Supplementary Table 1. SOD and CPO loading and activity

Enzyme	NGs	Added enzyme	Residual (U mL^{-1}, 2mL)	Loading (U)	Loading amount (U mg^{-1})	Activity
SOD	25 mg	2000 U	452.07	1095.86	43.83	76%
CPO	25 mg	1200 U	287.30	625.40	25.02	84%

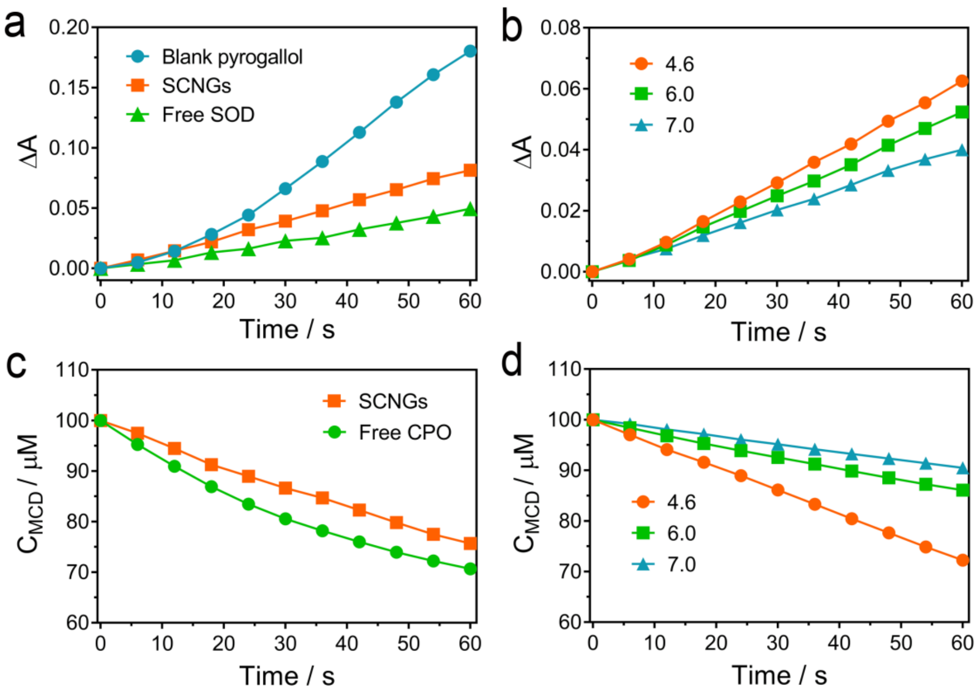

Supplementary Figure 5. The initial reaction rate of SOD (**a**) and CPO in SCNGs (**c**), and the activity tests of SOD (**b**) and CPO (**d**) in SCNGs at different pH (4.6, 6.0, 7.0).

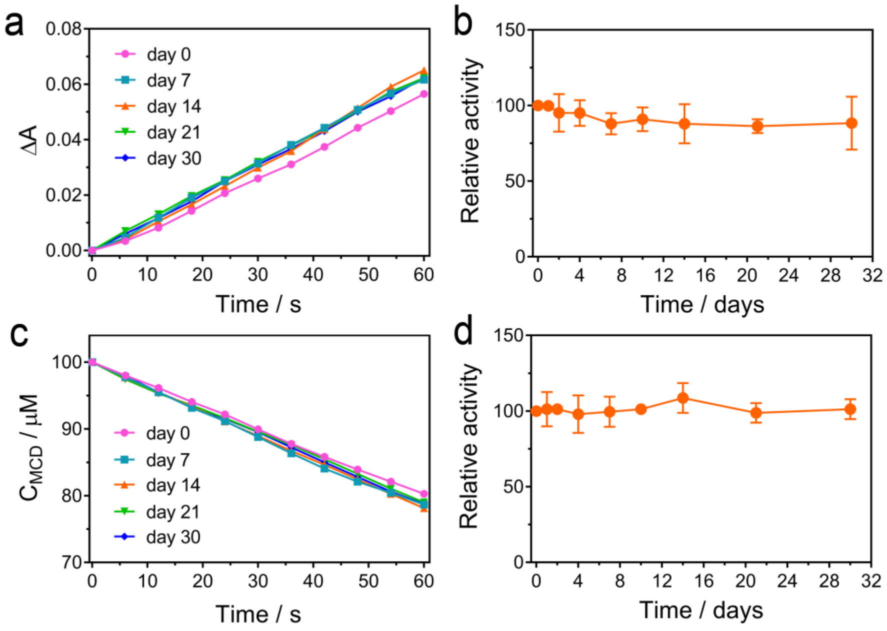

Supplementary Figure 6. The initial reaction rate of SOD (**a**) and CPO (**c**) in SCNGs for different storage days. The storage activity tests of SOD (**b**) and CPO (**d**) in SCNGs for 14 days. The experiment was conducted three times, and representative results are present.

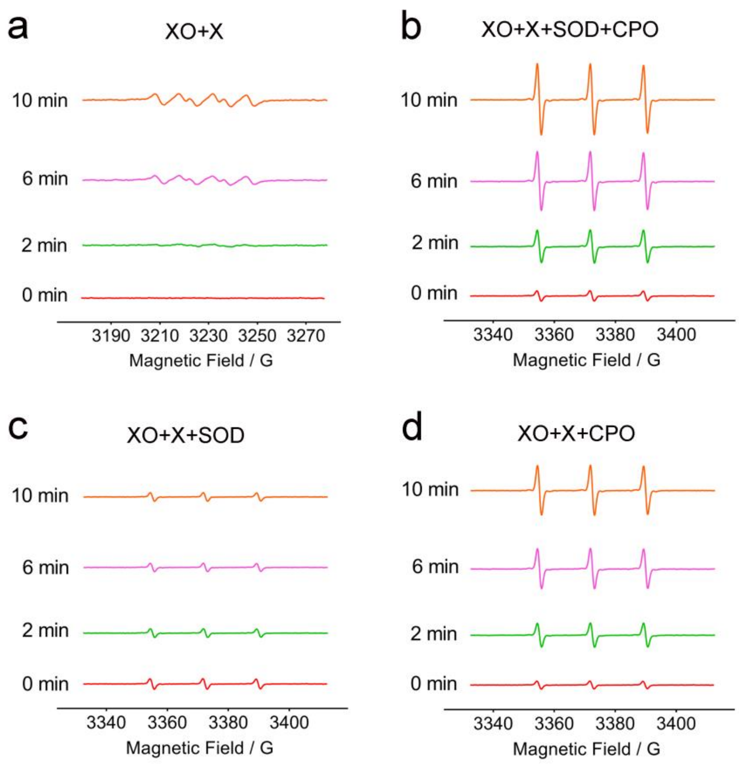

Supplementary Figure 7. Time-dependent EPR signals of XO and X in the presence of DMPO (**a**), and the free SOD/CPO (**b**), SOD (**c**) and CPO (**d**) in the presence of TEMP. EPR spectra were carried out in PBS buffer (20 m M, pH 6.8).

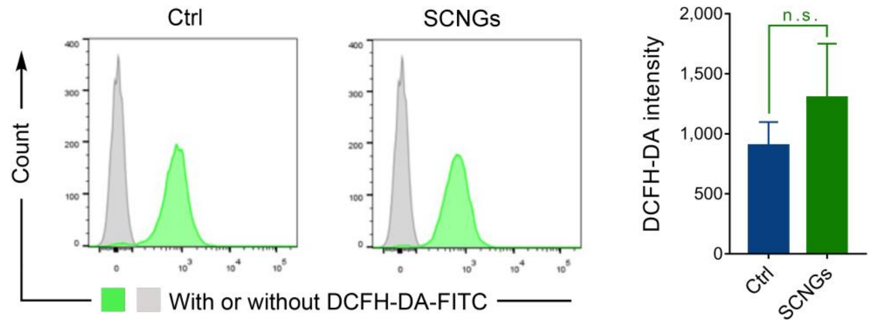

Supplementary Figure 8. The corresponding flow-cytometry analysis of HL-7702 cells treated with PBS and SCNGs at the IC_{50} value for 24 h using carboxy-H_2DCFDA as a ROS detector (left) and the corresponding data analysis (right). The experiment was conducted three times, and representative results are present. n.s. represents no significant differences.

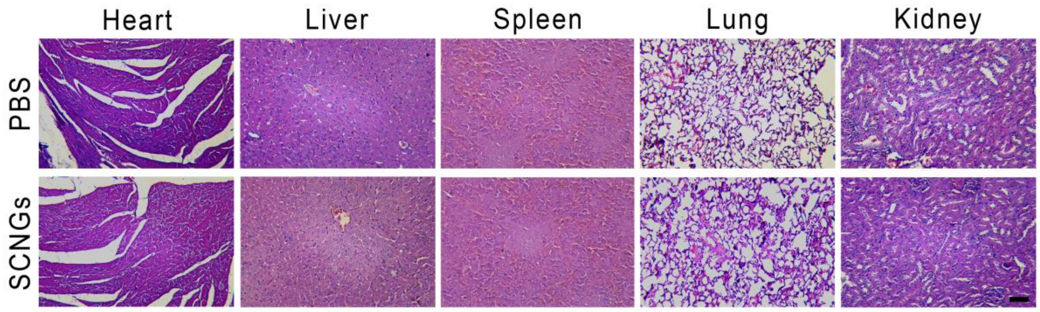

Supplementary Figure 9. H&E staining of the major organs of each group of HCC PDX mice after different treatments. Scale bar, 200 μm.

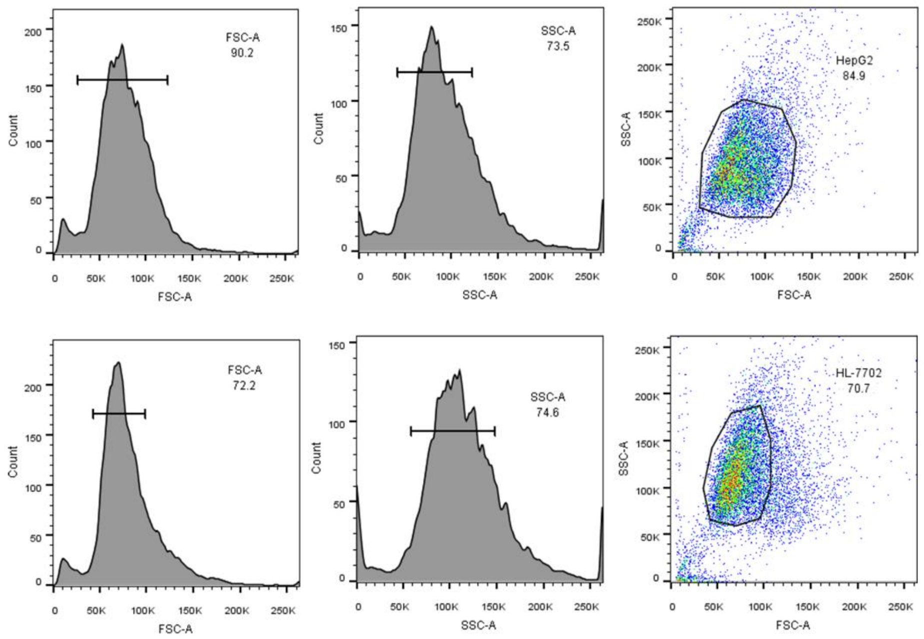

Supplementary Figure 10. Typical cytometry gating used.
